# The RGS-RhoGEFs control the amplitude of *YAP1* activation by serum

**DOI:** 10.1038/s41598-021-82027-4

**Published:** 2021-01-27

**Authors:** Brandon S. Lane, Brigitte Heller, Morley D. Hollenberg, Clark D. Wells

**Affiliations:** 1 Department of Biochemistry & Molecular Biology, Indiana University School of Medicine, Indianapolis, IN 46202 USA; 2 Department of Physiology & Pharmacology, Cumming School of Medicine, University of Calgary, Calgary, AB T2N 4N1 Canada; 3 Indiana University School of Medicine, John D. Van Nuys Medical Science Building. 635 Barnhill Dr., Rm. 4079A, Indianapolis, IN USA

**Keywords:** RHO signalling, Actin, Breast cancer, Growth factor signalling

## Abstract

Actin-dependent mechanisms drive the nuclear translocation of Yap1 to enable its co-activation of transcription factors that induce pro-growth and survival programs. While Rho GTPases are necessary for the nuclear import of YAP1, the relevant Guanine Exchange Factors (GEFs) and GTPase Activating Proteins (GAPs) that connect this process to upstream signaling are not well defined. To this end, we measured the impact of expressing sixty-seven RhoGEFs and RhoGAPs on the YAP1 dependent activity of a TEAD element transcriptional reporter. Robust effects by all three members of the regulator of G-protein signaling (RGS) domain containing RhoGEFs (ArhGEF1, ArhGEF11 and ArhGEF12) prompted studies relating their known roles in serum signaling onto the regulation of Yap1. Under all conditions examined, ArhGEF12 preferentially mediated the activation of YAP1/TEAD by serum versus ArhGEF1 or ArhGEF11. Conversely, ArhGEF1 in multiple contexts inhibited both basal and serum elevated YAP1 activity through its GAP activity for Gα_13_. The sensitivity of such inhibition to cellular density and to low states of serum signaling supports that ArhGEF1 is a context dependent regulator of YAP1. Taken together, the relative activities of the RGS-RhoGEFs were found to dictate the degree to which serum signaling promotes YAP1 activity.

## Introduction

The Hippo pathway integrates cell state cues to inhibit cell growth or to promote apoptosis. Such effects control organ size, tissue repair and stem cell identity as well as the suppression of diseases including cancer^1–3^. Activation of HIPPO signaling by anti-growth cues such as intercellular contacts and metabolic stresses converges onto the activation of the Large Tumor Suppressor 1/2 (LATS) kinases. Active LATs1/2 then phosphorylate Yes-Associated Protein (YAP1) and an associated Amot family adaptor protein^2^ to trigger YAP1 sequestration and/or degradation^3–6^. In parallel, a threshold level of actomyosin mediated cell tension is necessary for the nuclear translocation of YAP1^7–9^, a process that requires signaling by Rho family GTPases (Fig. S1A)^8–11^.

While Rho GTPases are essential for the nuclear translocation of YAP1, there are significant knowledge gaps regarding the upstream signaling that controls this process. Rho GTPases are activated by guanine exchange factors (GEFs) that promote GTP binding and inactivated by GTPase activating proteins (GAPs) that accelerate GTP hydrolysis^12,13^. Additional protein–protein and protein-lipid interaction motifs in both RhoGEFs and RhoGAPs contextualize their effects to specific signaling complexes^14,15^. Consequently, the identification of the GEFs and GAPs that regulate YAP1 may shed light on operative upstream signaling pathways. Here, a candidate expression screen identified all members of the Regulator of G-protein signaling (RGS) family of RhoGEFs (ArhGEF1/p115RhoGEF, ArhGEF11/PDZ-RhoGEF/GTRAP48 and ArhGEF12/LARG) in promoting YAP1/TEAD dependent transcription. Because the RGS-RhoGEFs activate RhoA in response to serum signaling^16,17^, we investigated their relative roles in controlling serum-signaling flux onto the regulation of YAP1. These studies utilized breast cancer cells in which all three RGS-RhoGEFs are co-expressed and regulate invasive behavior^18–20^. In multiple approaches, ArhGEF12 was determined to be preferentially required over ArhGEF11 for the activation of YAP1 by serum. Conversely, ArhGEF1 limited YAP1 activity in a manner that was sensitive to cell density.

## Results

### Identification of Rho GEFs and GAPs that regulate YAP1/TEAD-dependent transcription

**Figure 1:**
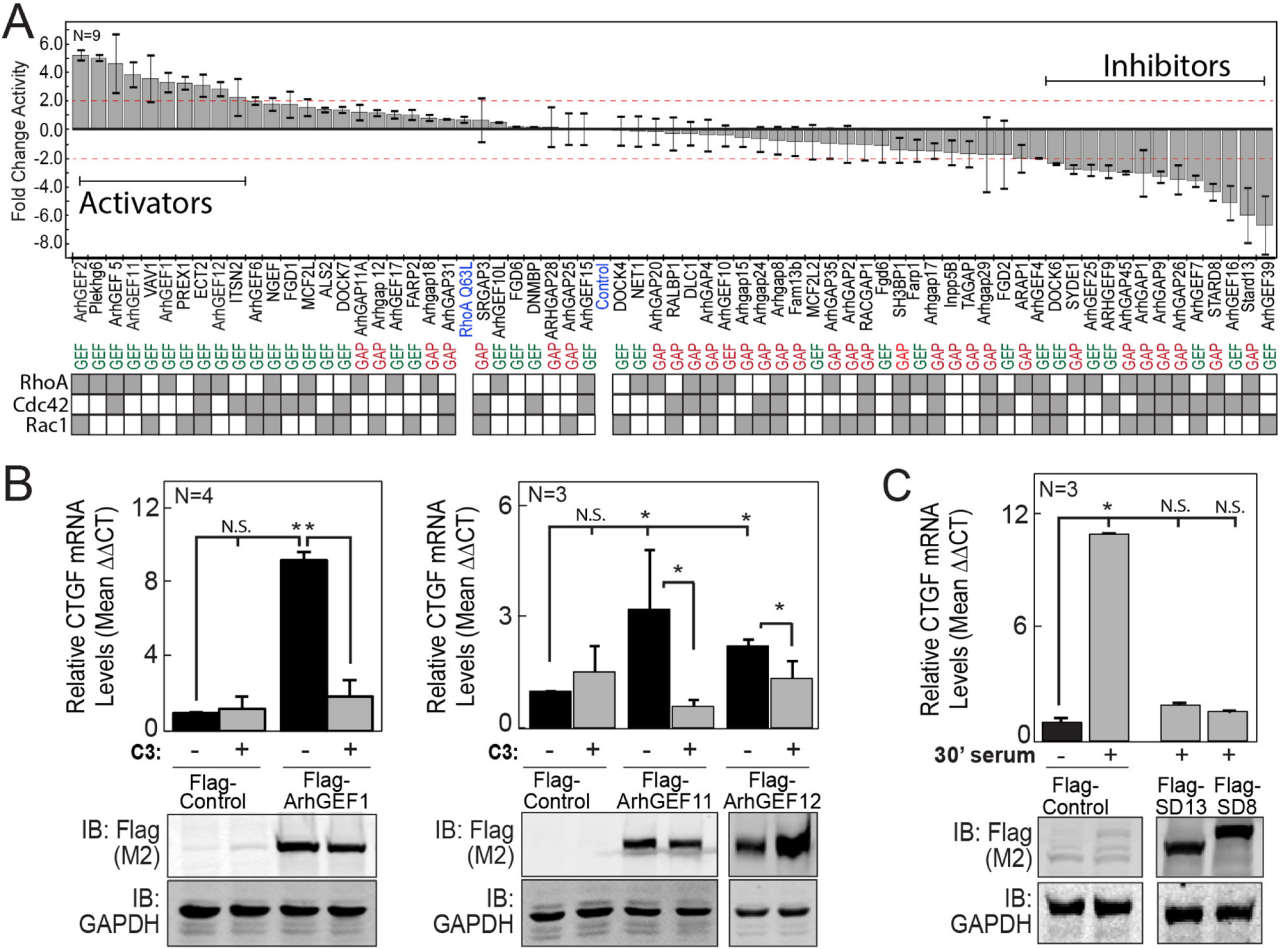
Candidate expression screen for Rho GEFs and GAPs that regulate YAP1-dependent transcription. (**A**) Mean fold-change in normalized TEAD reporter activity in BT474 cells expressing the indicated Flag-tagged RhoGEFs or RhoGAPs versus a Flag-control. Cells were also transfected with YAP1, the TEAD reporter and TK-Renilla (top panel). Activators and inhibitors mark twofold or greater effects. GEF (Green) or GAP (Red) domain identities are aligned with the genecard.org selectivity for RhoA, Rac1 and Cdc42 (bottom panel). (**B**) Fold-change in CTGF transcript levels as measured by qRT-PCR from cDNA synthesized from total mRNA isolated from MCF7 cells transfected for 48 h with plasmids expressing Flag control or a Flag-tagged RGS-RhoGEF in combination with control plasmid or a plasmid that expresses the C3 exotransferase (top panels). Immunoblot analysis of protein lysates from replicate samples using antibodies against the Flag-tag and GAPDH (bottom panels). (**C**) Fold-change in CTGF transcript levels from MCF7 cells transfected with a control plasmid or plasmids that express either the STARD13 (SD13) or STARD8 (SD8) RhoGAPs. Cells were cultured in serum free media for 24 h and then treated for 30 m with media containing either 0% (−) or 10% (+) fetal bovine serum. Data represent mean values, error bars show standard deviation of mean. Variability unless indicated otherwise was queried by one-way ANOVA. *p-value < 0.05 from contrasts identified by the Bonferroni Multiple Analysis Test; N.S. = non-significant contrasts of interest. Raw images for all immunoblots are in Figs. S6 and S7.

Sixty-seven RhoGEFs and RhoGAPs were screened for their relative abilities to modulate the transcriptional activity of a YAP1/TEAD dependent reporter assay. To enhance the dynamic range of YAP1 dependent activation and inhibition, multiple parameters of the assay were optimized including cell type, cell density, length of time after transfection and the amount of co-transfected Yap1 (Fig. S1B–E). BT474 cells, a hormone negative breast cancer cell line, were eventually selected for these assays due to their dependence on Yap1 co-transfection for reporter activity. The average fold-differences in TEAD reporter activity in response to expression of 36 RhoGEFs and 31 RhoGAPs were reported alongside their published activities for RhoA, Rac1 and Cdc42 (Fig. 1A). Targeted validation of screen results evaluated the effects of the RGS-RhoGEFs and Deleted in Liver Cancer (DLC) members on CTGF transcript levels, a common endogenous surrogate for Yap1 activity^21^. In concordance with screen results, CTGF transcript levels increased upon expression of each of the RGS-RhoGEFs while it decreased in cells in which DLC2/STARD13/ArhGAP37 or DLC3/STARD8/ARHGAP38 were expressed relative to controls (Fig. 1B,C). The loss of CTGF induction by the RGS-RhoGEFs upon co-expression of the C3 transferase exotoxin (an inhibitor of RhoA) confirmed a requirement for RhoA for their effects.

### Validation of CTGF transcription as an endogenous surrogate measure of YAP1/TEAD activity

**Figure 2:**
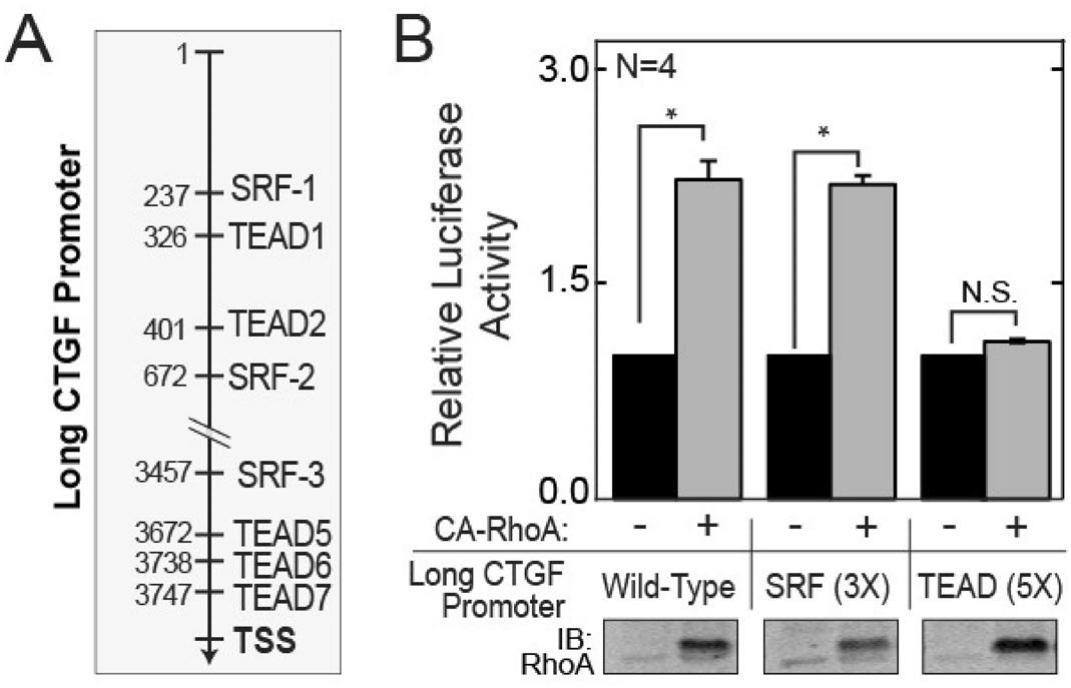
Induction of CTGF expression by RhoA Requires TEAD but not SRF elements. (**A**) Depiction of the SRF and TEAD elements in the long (~ 3.9 kb) CTGF promoter. (**B**) Fold-change in relative luciferase activity in lysates from MCF7 cells transfected with a PGL3.1 reporter containing the indicated variants of the 4 kb (long) promoter of CTGF in combination with Thymidine Kinase (TK)-Renilla and either a control plasmid or a plasmid expressing the constitutively active (CA)-RhoA (Q63L) mutant.

While CTGF transcript levels are often used to infer endogenous YAP1/TEAD activity, both SRF^22^ and TEAD^21^ directly bind and activate the CTGF promoter. Further, F-actin formation promotes MRTF to enter the nucleus where it forms a complex with SRF that directly binds and activates the CTGF promoter^23,24^. We therefore compared the relative importance of SRF elements (SRE) versus TEAD elements in the CTGF promoter for transcriptional activation by RhoA. For this purpose, transcriptional reporters were constructed containing fragments of the wild-type CTGF promoter consisting of a “short” 700 bp sequence immediately upstream of the transcriptional start site (TSS) and a “long” version that includes the “short” sequence and 3.2 kb of upstream sequence that contains two additional SRF and TEAD elements. Both long and short promoters were analyzed to address the contexts in which the TEAD elements and SRF elements were previously studied^21,23^. Mutant versions of the two CTGF promoters that lack all Serum Response Elements (SRE) or the TEA elements were then created (Figs. 2A, S2A,B) and cloned into a pGL3.1 reporter plasmid. Analysis of these reporters by dual luciferase assay revealed that the loss of SRF elements (long 3X-SRF and short 1X-SRF) did not reduce transcriptional activation by constitutively active RhoA (Q63L) in comparison to the wild-type counterparts (long WT and short WT). However, inactivation of TEAD binding sites (long 5X-TEAD or 3X-TEAD) resulted in a complete loss of activation by RhoA (Q63L) (Figs. 2B, S2C). The essentiality of endogenous YAP1 for this process was supported by the inhibition of CTGF induction by serum in cells treated with Verteporfin (Fig. S2D), a small molecule inhibitor of YAP1^25^. Taken together, CTGF transcript levels are indicated to be a specific surrogate measure of YAP1/TEAD activation by RhoA.

### ArhGEF12 preferentially transmits serum signaling to activate YAP1/TEAD

**Figure 3:**
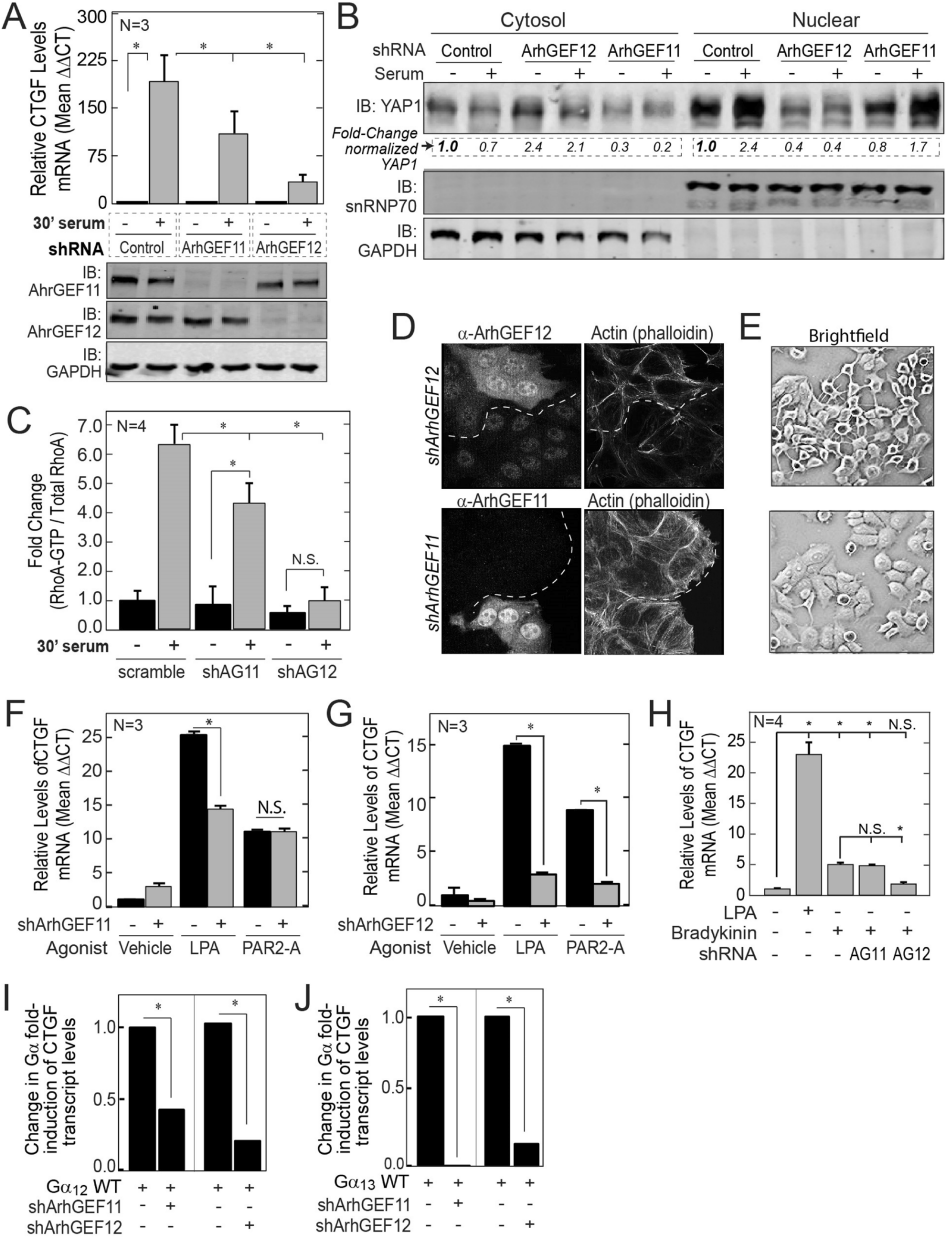
ArhGEF12 is required for serum signaling to RhoA and for YAP1 activation. (**A**) MCF7 cells transduced with lentivirus encoding short hairpin (sh) control, shArhGEF11, or shArhGEF12 and treated with media for 30 min containing 0% or 10% serum. Fold-change in relative CTGF transcript levels (Top Graph). Immunoblot (IB) analysis of ArhGEF11, ArhGEF12 and GAPDH (bottom panels). (**B**) Immunoblot analysis measuring YAP1, snRNP70 and GAPDH protein levels in nuclear and cytosol fractions prepared from MCF7 cells described in A. (**C**) Ratios of RhoA-GTP and total RhoA levels were measured by G-LISA, Cytoskeleton, Inc. from lysates from MCF7 cells with shcontrol, shArhGEF11 or shArhGEF12. Cells were serum-starved for 24 h before treatment with 0% or 10% serum for 30 min. (**D**) MCF7 cells stably transduced with shRNA control were mixed with cells transduced with either shArhGEF11 or shArhGEF12 and then immunostained with antibodies against ArhGEF11 or ArhGEF12 in combination with phalloidin-CruzFluor 594. Dashed lines demarcate cell clusters with normal expression versus cells silenced for either GEF. (**E**) Brightfield images of MCF7 cells silenced for ArhGEF11 (Bottom Left) or ArhGEF12 (bottom Right). (**F**, **G**) Fold-change in CTGF transcript levels induced by LPA (1 µM) or the PAR2-A peptide (5 µM) in serum starved MCF7 cells stably transduced with either shControl (−) or shArhGEF11 (in G) or shArhGEF12 (in H). (**H**, **I**) fold-change in the induction of CTGF transcript by Gα_12_ (in J) or Gα_13_ (in K) in MCF7 cells transduced with shControl (−), shArhGEF11 or shArfGEF12. (**J**) Fold-change in CTGF transcript levels induced by LPA (1 µM) or Bradykinin (10 nM) in MCF7 cells transduced with shControl, shArhGEF11 (shAG11) or shArhGEF12 (shAG12). In I/J, *p-value of < 0.05 computed by student t-test.

Serum mitogens such as lysophosphatidic acid (LPA) and coagulation proteases agonize G-protein coupled Receptors (GPCR) to stimulate nucleotide exchange on the Gα subunit of the G_12/13_ family of heterotrimeric G-proteins^26–28^. The GTP bound forms of Gα_12_ and Gα_13_ then bind and enhance the GEF activity of the RGS-RhoGEFs for RhoA^16,29,30^. The duration of this complex is simultaneously limited by the GAP activity of the RGS domains for the bound Gα subunit^29,31^. While this bi-directional signaling mechanism is well-established in vitro, the relative degree to which each RGS-RhoGEF transmits versus inactivates serum signaling onto YAP1 in cells is poorly understood. For this purpose, YAP1 activation by serum was defined in both MCF7 and BT474 cells stably transduced with lentiviruses encoding either a short-hairpin (sh) control, shArhGEF11 or shArhGEF12 (Fig. 3A). ArhGEF11 silencing in MCF7 cells resulted in an ~ 40% reduction in CTGF induction by serum, but its silencing in BT474 cells had no effect on this process. In contrast, ArhGEF12 silencing reduced the induction of CTGF by serum by ~ 85% in MCF7 cells and by ~ 50% in BT474 cells (S3A-D). However, the relative levels of phosphorylated S127-Yap1 in lysates from cells silenced for ArhGEF11 or ArhGEF12 were similar to control cells (Fig. S3A, C). This suggests that both GEFs primarily regulate YAP1 through RhoA as it may regulate the nuclear import of YAP1 independently of its phosphorylation state^8^. To further investigate YAP activation, the redistributions of YAP1 from the cytosol to the nucleus in response to serum were compared between control cells and ArhGEF11 or ArhGEF12 silenced cells (Fig. 3B). Under serum starvation, the levels of YAP1 were lower in nuclear fractions from ArhGEF12 silenced cells versus control or ArhGEF11 silenced cells. ArhGEF12 silenced cells also did not show the shift of YAP1 levels from the cytosol to the nucleus after serum treatment that was observed for control and ArhGEF11 silenced cells. In concordance with YAP1 activation by serum, ArhGEF12 silenced cells also did not experience an increase in relative RhoA-GTP levels after serum treatment. In contrast, relative RhoA-GTP levels were increased by sixfold after serum treatment in control cells and by fourfold in ArhGEF11 silenced cells (Fig. 3C). Imaging further showed that ArhGEF12 silenced cells had lower levels of phalloidin stained F-actin and were highly rounded in relation to control cells or ArhGEF11 silenced cells (Fig. 3D,E).

Given the differential requirements of ArhGEF11 and ArhGEF12 for serum initiated signaling, we investigated the degree to which this could be explained by their participation in specific receptor mediated signaling complexes. While serum mitogens such as lysophosphatidic acid (LPA) and thrombin activate both G_12_ and G_13_ coupled receptors^32,33^, LPA dependent signaling is reported to selectively use ArhGEF11 for RhoA activation, whereas protease activated receptor (PAR) signaling was found to specifically utilize ArhGEF12^17^. We therefore investigated the degree to which these serum agonists act specifically through ArhGEF11 or ArhGEF12 to activate YAP1. MCF7 and BT474 cells silenced for ArhGEF11 showed reduced CTGF induction by LPA but not by Par2-a (a peptide agonist for type-2 PARs) versus control cells (Fig. 3F, Fig. S3E). In contrast, silencing of ArhGEF12 resulted in an ~ 80% loss in CTGF induction by both agonists in both cell types (Figs. 3G, S3F). Given this high level of dependence on ArhGEF12 for YAP1 activation by both agonists, we investigated if ArhGEF12 may also be required for the activation of YAP1 by Bradykinin, which acts on Gα_q_ and not G_12/13_ coupled receptors^34^. In comparison to LPA, bradykinin weakly induced CTGF transcript levels. Further, the induction of CTGF by Bradykinin was inhibited upon silencing of ArhGEF12 but not ArhGEF11 (Fig. 3H). Because Bradykinin acts through the RhoGEF Trio^35^, the effects of ArhGEF12 are likely indirect. The constitutive loss of F-actin levels and the cell rounding in ArhGEF12 silenced cells suggests that ArhGEF12 may tonically stimulate RhoA to enable weak agonists such as bradykinin to cross a threshold of actomyosin activation that is required for the nuclear import of YAP1^8^.

The relative roles of ArhGEF11 and ArhGEF12 in Gα_12_ versus Gα_13_ signaling to YAP1 were then queried. To isolate the effects of silencing ArhGEF11 or ArhGEF12 on YAP1 activation by either Gα subunit, the fold-inductions of CTGF levels by Flag-tagged Gα_12_ or Flag-tagged Gα_13_ versus a flag-control were determined in the backgrounds of an shcontrol, shArhGEF11 and shArhGEF12 (Fig. S3G). Silencing of either ArhGEF11 or ArhGEF12 resulted in a similar inhibition in the fold-activation of CTGF induction by either Gα_12_ or Gα_13_ (Fig. 3I,J). Both GEFs therefore appear to have a similar capacity to mediate signaling by these GTPases. The preferential effects observed for ArhGEF12 in serum signaling may therefore be highly sensitive to stoichiometric changes in the levels of these GTPases.

### ArhGEF1 limits the activation of YAP1 by serum under specific cellular contexts

**Figure 4:**
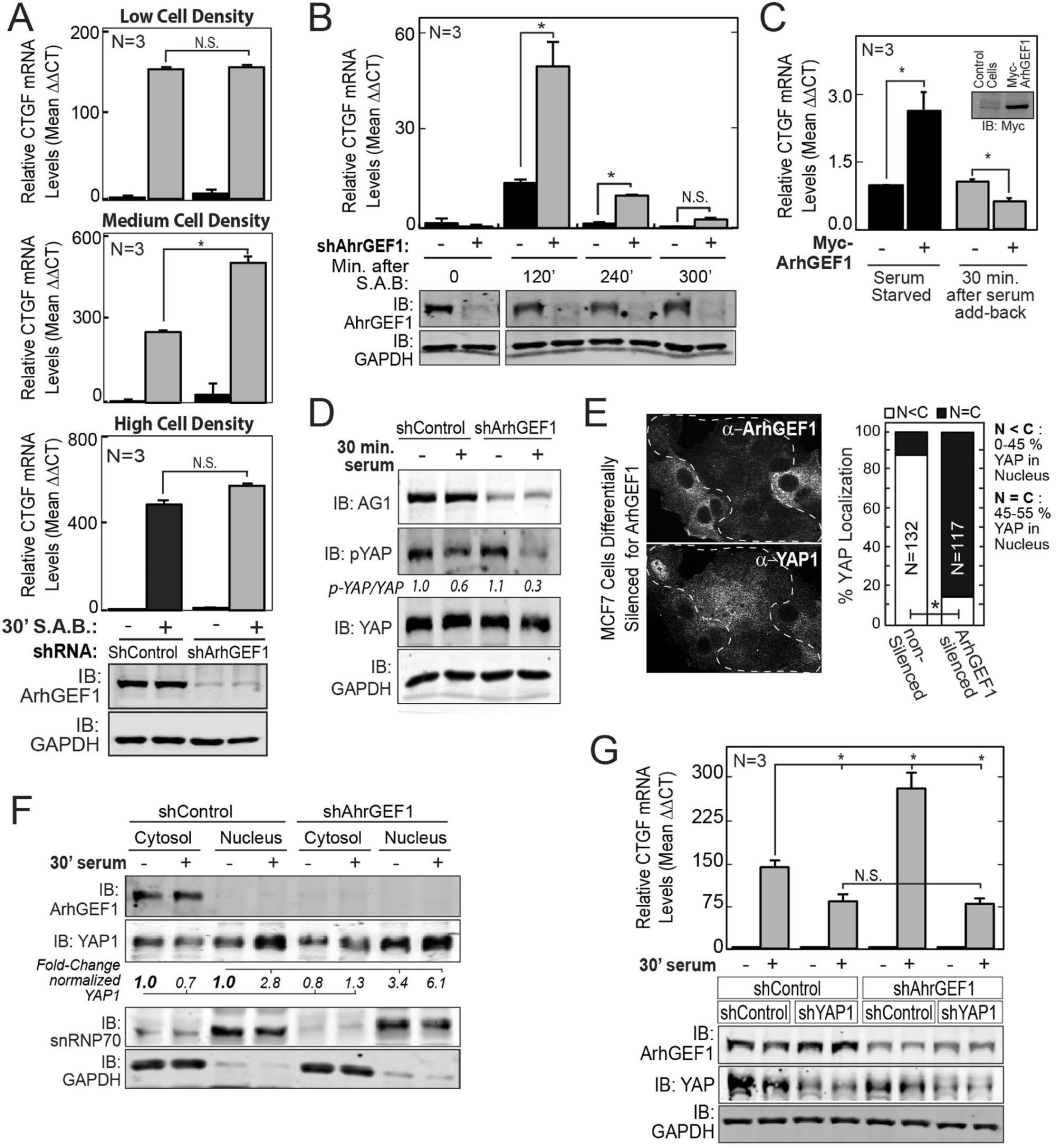
ArhGEF1 limits the activation of YAP1 by serum. (**A**) MCF7 cells transduced with shControl or shArhGEF1 were grown at low (< 20%), intermediate (40–70%) or high (> 95%) density. After 24 h of serum starvation, media with 0% or 10% serum was added back (S.A.B.) for 30 min. Fold-change in CTGF transcript levels were measured by qRT-PCR (Top 3 Graphs). ArhGEF1 and GAPDH protein levels in low density cells were analyzed by immunoblot (bottom 2 panels). (**B**) Control and ArhGEF1 silenced MCF7 were serum starved before treatment with 10% serum for the indicated times. Fold-change in CTGF transcript levels were measured by qRT-PCR (Top Graph) and relative ArhGEF1 and GAPDH protein levels were detected by immunoblot analysis (Bottom Panels). (**C**) MCF7 cells stably expressing Myc-tag control or Myc-ArhGEF1 were grown without serum for 24 h and then in media with 0% or 10% serum. Fold-change in CTGF transcript levels was measured by qRT-PCR and relative levels in myc-tagged protein was measured by immunoblot (inset). (**D**) Immunoblot analysis with antibodies against phospho-S127 YAP1, total YAP1 and GAPDH was performed on lysates from MCF7 cells transduced with shRNA control or shArhGEF1 lentivirus after serum starvation for 24 h and then treatment with media with 0% or 10% serum for 30 min. The ratio of p-YAP1/total-YAP1 pixel intensities are given below second panel. (**E**) MCF7 cells with differential silencing of ArhGEF1 were fixed and immunostained for ArhGEF1 (top panel) and YAP1 (middle panel). Dashed line separates ArhGEF1 silenced and non-silenced cells. A quantitation of the frequency of YAP1 localization in nucleus and cytosol was undertaken (Graph, at right). (**F**) An immunoblot analysis with antibodies against ArhGEF1, YAP1, snRNP70 and GAPDH from Cytosol and nuclear fractions prepared from MCF7 cells with either control or ArhGEF1 silencing. Cells were serum starved for 24 h before treatment with 0 or 10% serum for 30 min. Fold-change in relative YAP1 levels (versus controls) in the cytosol and in nucleus (below second panel). (**G**) Serum starved MCF7 cells silenced for ArhGEF1 alone or in combination with YAP1 were treated with media with 0 or 10% serum for 30 min. Fold-change in CTGF transcript levels were measured by qRT-PCR (top panel). Immunoblot analysis from replicate samples with antibodies against ArhGEF1, YAP1 and GAPDH (Bottom three Panels). In (**E**) ***p-value < 0.0001 computed by 3 × 3 Fischer exact test.

Unlike silencing ArhGEF11 or -12, the induction of CTGF by serum was significantly higher in cells stably silenced for ArhGEF1 if cells were growing at intermediate density (Fig. 4A). The relative effects of silencing ArhGEF1/11/12 by siRNA on CTGF induction by serum was also directly compared. In consonance with results from cells treated with shRNAs, cells silenced for ArhGEF1 using siRNA had increased serum induction of CTGF, whereas siRNA treated cells against ArhGEF12 and to a lesser extent for ArhGEF11 had reduced levels of CTGF induction by serum (Fig. S4A,B). Further, the sensitivity to low and high cells density of the activating effects of silencing ArhGEF1 on CTGF induction by serum were validated in MCF7 cells silenced for ArhGEF1 with siRNA (Fig. S4C). In concordance with results in MCF7 cells, MDA-MB-231 cells silenced for ArhGEF1 also showed significantly increased fold-induction of CTGF by serum over non-silenced cells (Fig. S4D). However, serum had a reduced effect on CTGF induction in BT474 cells silenced for ArhGEF1 (Fig. S4E). Given the effects of cell-density on ArhGEF1 activity in MCF7 cells, this difference may be due to the proclivity of BT474 cells to form compact colonies of attached cells within hours of plating. Further, enhancement of CTGF levels in ArhGEF1 silenced cells was greatest after 2 h of serum treatment, but declined at four-hours and was not evident by 5-h (Figs. 4B, S4F). To further investigate the effects of serum on ArhGEF1 action, the fold-change in CTGF levels after serum treatment was examined in cells overexpressing ArhGEF1 relative to control cells. Under growth conditions without serum, cells with ArhGEF1 overexpression had increased levels of CTGF transcript versus control cells. Whereas, cells overexpressing ArhGEF1 had reduced induction of CTGF by serum (Figs. 4C, S4G). In summary, both silencing and overexpression experiments find that ArhGEF1 limits the magnitude of “burst” signaling that activates Yap1 upon the add-back of serum to cells in a manner that is sensitive to cell-density. However, ArhGEF1 overexpression may promote YAP1 activity in cells experiencing states of low serum signaling or that are desensitized from extended serum exposure.

The regulation of YAP1 activity by ArhGEF1 was then investigated in more detail. Immunoblot analysis found a reduced ratio of S^127^-phosphorylated YAP1 over total YAP1 in ArhGEF1 silenced cells. ArhGEF1 may therefore inhibit a target that in turn inhibits YAP1 phosphorylation by LATs1/2 (Fig. 4D). YAP1 activation upon ArhGEF1 silencing was further demonstrated by fluorescence based imaging that shows an increased distribution of YAP1 in the nuclei of ArhGEF1 silenced MCF7 cells grown at intermediate density in comparison to non-silenced cells (Fig. 4E). However, YAP1 localization was not affected by ArhGEF1 silencing in cells growing at very high or low density (Fig. S4H–J). Imaging results were confirmed by a biochemical analysis showing increased levels of total YAP1 in nuclear fractions from ArhGEF1 silenced cells versus non-silenced cells (Fig. 4F). The degree to which ArhGEF11 or ArhGEF12 are required for the enhanced signaling seen upon ArhGEF1 silencing was then investigated. Surprisingly, cells silenced for ArhGEF11 or ArhGEF12 displayed a similar loss in CTGF induction in a background in which ArhGEF1 was also silenced (Fig. S4K). In confirmation that the target(s) of ArhGEF1 inhibition signal through Yap1, the fold-induction of CTGF by serum was similar between cells silenced for YAP1 alone and cells silenced for YAP1 in combination with ArhGEF1 (Figs. 4G, S4L).

**Figure 5:**
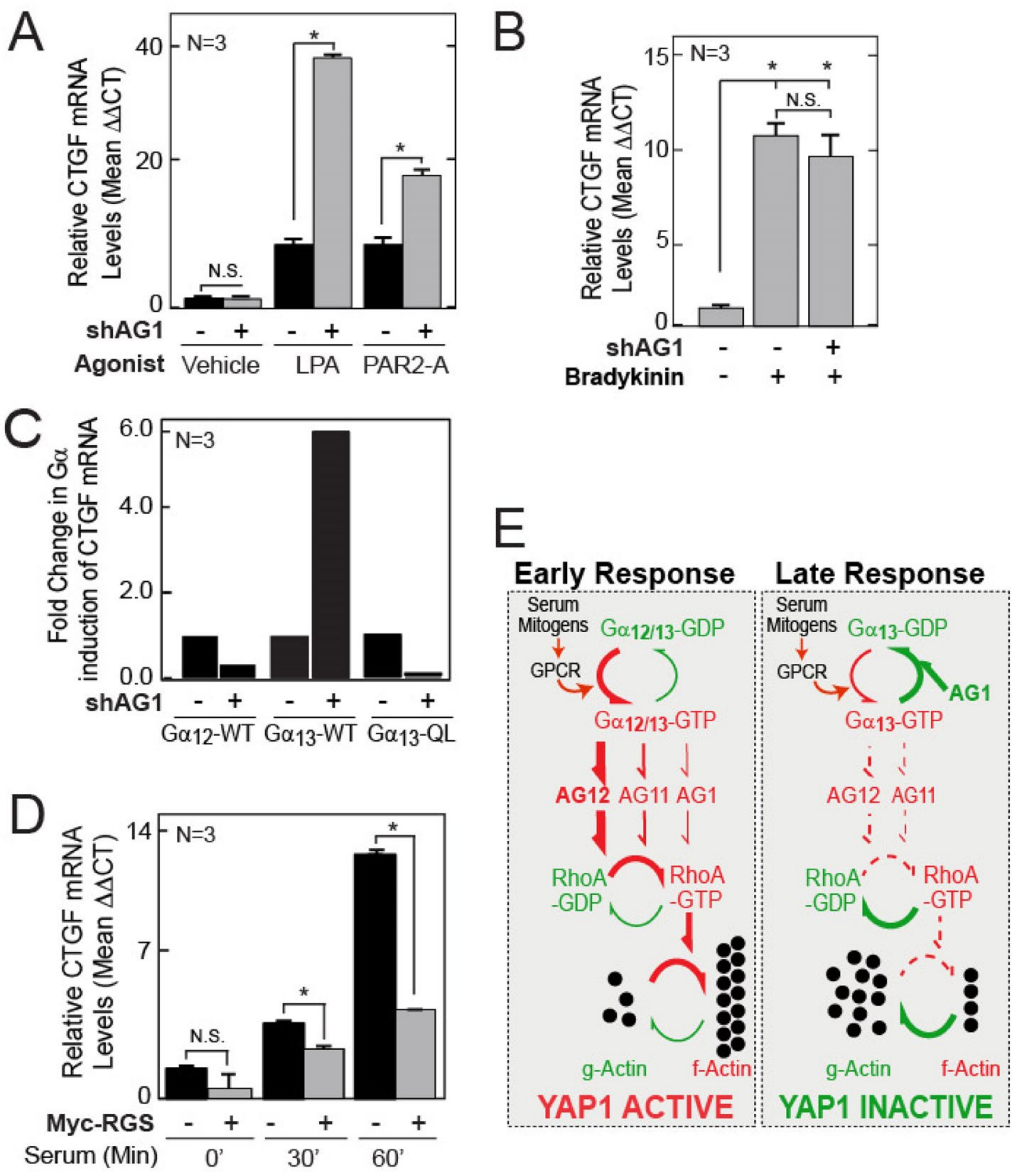
ArhGEF1 selectively inhibits Gα_13_ to reduce the activation of Yap1 dependent transcription by serum. (**A**) Serum starved MCF7 cells transduced with control or shRNA against ArhGEF1 were treated for 30 min with vehicle, LPA (1 µM), or the PAR2-A peptide (5 µM). Fold-change in CTGF transcript levels was then determined using qRT-PCR. (**B**) MCF7 cells stably transduced with shControl or shArhGEF1 (shAG1) were treated for 30 min with vehicle or Bradykinin (10 nM). Fold-change in CTGF transcript levels were then measured by qRT-PCR (**C**) Fold induction of CTGF transcript in MCF7 cells infected with lentivirus encoding either shControl (−) or shArhGEF1 (shAG1) in combination with lentivirus that expresses either Flag-Control, Gα_12_-WT, Gα_13_-WT or the GTPase deficient Gα_13_-QL mutant. (**D**) Fold-change in CTGF transcript levels as measured by qRT-PCR from MCF7 cells expressing control or myc-tagged ArhGEF1-RGS. Before lysis, cells were serum starved for 24 h and then treated with media alone or media containing 10% FCS for 60 min. (**E**) Model for the combined activities of the RGS-RhoGEFs in serum signaling to YAP1.

The intracellular targets of ArhGEF1 inhibition were then characterized. Similar to observations with whole serum, CTGF inductions by both LPA and the Par-2a peptide were more efficacious in ArhGEF1 silenced cells versus non-silenced cells (Figs. 5A, S5A). In contrast, ArhGEF1 silencing did not affect CTGF induction by Bradykinin (Fig. 5B). This fits with the in vitro selectivity of the RGS domain of ArhGEF1 as a GAP for Gα_12/13_ but not for Gα_q_^29,31^. Consequently, further experiments focused on the effects of ArhGEF1 on the induction of CTGF by expression of Flag-tagged versions of Gα_12,_ Gα_13_, or the Gα_13_ (QL) mutant that is GTPase deficient^36^ (Fig. 5C). In relation to non-silenced MCF7 cells, ArhGEF1 silenced cells experienced a greater fold-induction of CTGF upon expression of Flag-tagged Gα_13_, but a lower fold-induction of CTGF by Flag-tagged Gα_13_ (QL) or Flag-tagged Gα_12_. ArhGEF1 is therefore likely to specifically inhibit signaling by Gα_13_. Further, such inhibition is most likely due to its GAP activity as ArhGEF1 silencing did not increase CTGF induction by the Gα_13_ (QL) mutant. The loss of CTGF induction by both Gα_13_ (QL) and Gα_12_ in ArhGEF1 silenced cells is consistent with endogenous ArhGEF1 being activated by these GTPases in control cells. The dominant effects of increasing the GAP activity for Gα_13_ on serum signaling was further demonstrated by the inhibition of CTGF and Cyr61 (also a TEAD target^21^) induction by serum in cells that overexpress the ArhGEF1-RGS domain (Figs. 5D, S5B–D). A model summarizing the coordinated actions of the RGS-RhoGEFs in serum signaling onto YAP1 is presented in Fig. 5E.

**Figure 6:**
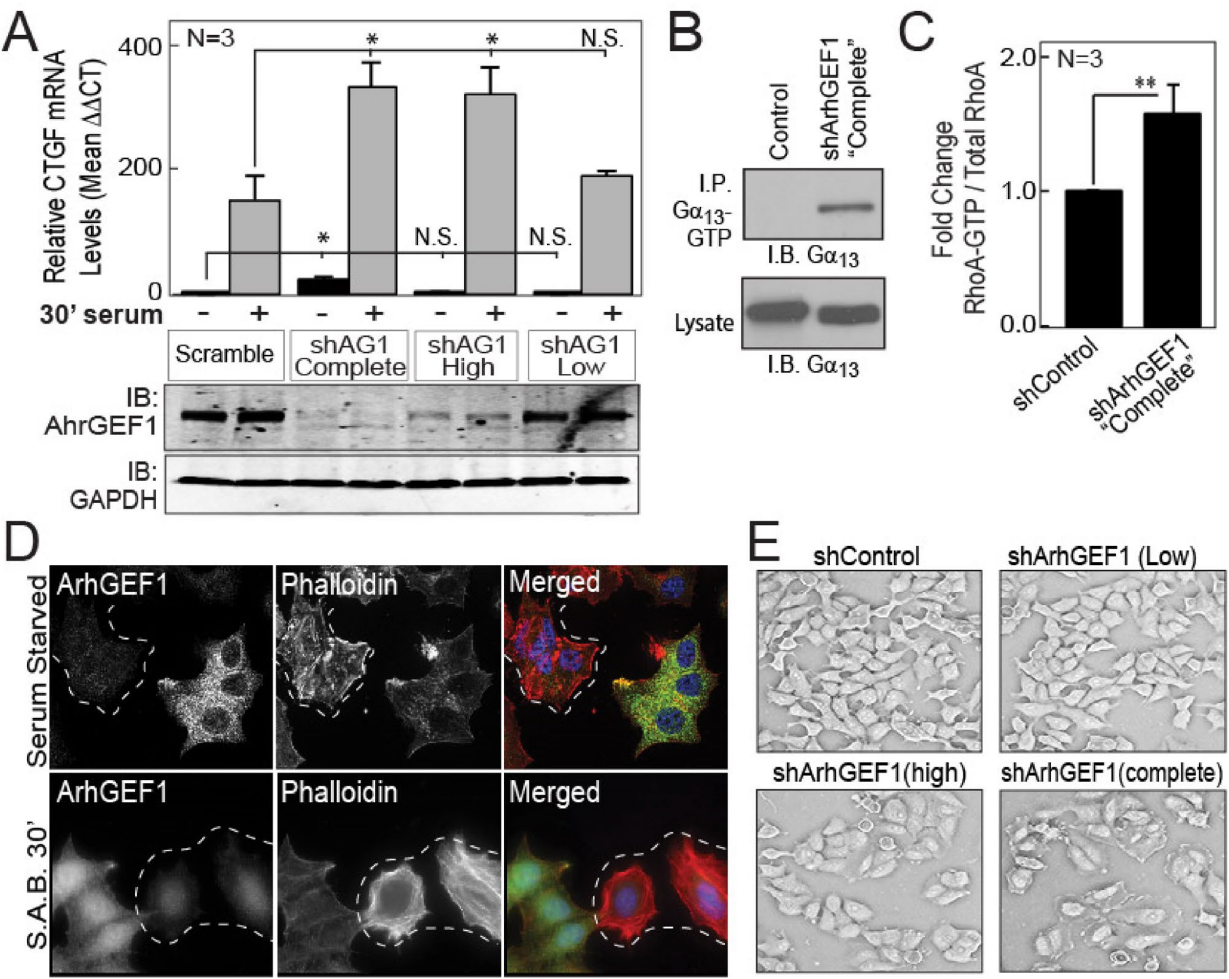
Complete ARHGEF1 silencing results in the basal activation of YAP1 and RhoA as well as enhanced actin fiber accumulation and cell flattening. (**A**) Serum Starved MCF7 cells with shcontrol, complete, high or low levels of ArhGEF1 (AG1) silencing were treated with 0 or 10% serum for 30 min. Fold-change in CTGF transcript levels were measured by qRT-PCR (top panel). Immunoblot analysis of ArhGEF1 and GAPDH levels of replicate samples (lower Panel). (**B**) Gα_13_-GTP was selectively immunoprecipitated with an anti-Gα_13_-GTP antibody from lysates from control and ArhGEF1 silenced cells. Gα_13_-GTP and total Gα_13_ were detected by immunoblot analysis of lysates and immunoprecipitations. (**C**) Total and GTP bound RhoA levels were measured by G-LISA (Cytoskeleton Inc.) in lysates from control or complete ArhGEF1 silenced MCF7 cells. Mean ratios from three biologic replicates are presented. (**D**) Immunofluorescence images of MCF7 cells with high and low ArhGEF1 silencing that were serum starved for 24 h before treatment with 0% or 10% serum (S.A.B.) for 30 min. Cells were stained for ArhGEF1, Phalloidin to visualize actin and DAPI to see nuclei. Dotted lines demarcate clusters of cells with high levels of ArhGEF1 silencing. At right, merged images of ArhGEF1 (green), Phalloidin (Red), and nuclei (DAPI stain). (**E**) Brightfield images of the MCF7 cell pools described in A growing continuously in media with 10% serum. In C, **p-value of < 0.01 computed by student t-test.

While ArhGEF1 was found to inhibit serum induced signaling, it was unclear if it also suppressed the basal activation of RhoA by Gα_13_. To answer this question, MCF7 cells were transduced with shRNA against ArhGEF1 and clonal pools were obtained with low (< 60%), high (80–90%) or complete (undetectable) levels of ArhGEF1 silencing. Analysis of CTGF induction by serum revealed that cells with high or complete ArhGEF1 silencing showed a similarly increased response versus control cells. However, basal CTGF transcript levels were specifically higher in cells with complete ArhGEF1 silencing in relation to control cells (Fig. 6A). In concordance with this being due to a lack of inactivation of Gα_13_, immunoblot analysis measured increased levels of Gα_13_-GTP in immunoprecipitations from cells with complete ArhGEF1 silencing versus control cells (Fig. 6B). Such higher Gα_13_ activity was accompanied by increased levels of RhoA-GTP (Fig. 6C) and a dramatic increase in stained F-actin (Fig. 6D). This increased Gα_13_ signaling may also explain why ArhGEF1 silenced cells persistently maintain a flattened morphology and fail to form a “typical” cobblestone monolayer (Fig. 6E).

## Discussion

This study relates the bi-directional control of the serum-signaling network by the RGS-RhoGEFs to the regulation of YAP1. In all breast cancer cell lines examined, ArhGEF12 was found to be the primary RGS-RhoGEF for transmitting serum signaling. Further, cells silenced for ArhGEF12 showed a profound loss of F-actin and a distinctly rounded morphology regardless of their serum exposure state. Conversely, ArhGEF1 inhibited this network via its specific GAP activity for Gα_13_. Such inhibitory activity was shown to limit the magnitude of serum signaling in a manner that was sensitive to cell density. ArhGEF1 was also shown to suppress basal Gα_13_ activity, RhoA activity and F-actin accumulation that likely explained the higher Yap1 activity and flattened morphology of these cells. Thus, ArhGEF1 is essential for both limiting the upper limit for serum signaling and for preventing constitutive Gα_13_ activity that otherwise likely dominantly dictates RhoA dependent processes. The sensitivity of the inhibitory effects of ArhGEF1 to cell density further suggests that it only reduces serum initiated signaling under specific cellular contexts. Together, ArhGEF12 and ArhGEF1 therefore define both the lower and upper ranges over which serum dependent signaling may affect RhoA and YAP1 activation.

Several RhoGEFs and RhoGAPs in an expression screen were found to regulate YAP1/TEAD activity. The proclivity of hits identified by this screen for activity towards RhoA is consistent with previous work implicating RhoA as the predominant Rho family GTPase in the regulation of YAP1. However, the lack of effects on TEAD activity by some RhoA selective factors including ArhGAP18, ArhGEF10L, ArhGEF15, NET1, ArhGAP20, RalBP1 and DLC1 supports that not all regulators of RhoA signal to YAP1/TEAD. Alternatively, the lack of an effect by these factors may be due to artifacts of overexpression such as mislocalization and non-physiologic stoichiometry. However, such effects are not likely common as many screen “hits” agree with previous studies. For instance, ArhGEF2, the top activator in the TEAD screen, was previously shown to activate YAP1^37^. Conversely, both ArhGEF7^38^ and STARD13^39^ were confirmed to inhibit YAP1. Importantly, screen results also suggest novel regulation of YAP1. For instance, the inhibition of YAP1/TEAD by five CDC42 selective RhoGEFs suggests that CDC42 may inhibit YAP1/TEAD. In addition, the activation of TEAD by ArhGEF5 may explain its ability to enhance the proliferation and the malignant properties of breast^40^ and lung^41^ cancer cells. Conversely, the cell rounding effects of the DLCs^42^ fit with the inhibition of the TEAD reporter activity by DLC2 and DLC3. Interestingly, while Ect2 has been found to regulate cytosolic YAP1 during cytokinesis^43,44^, here it is suggested to also promote canonical nuclear YAP1 activity.

Bi-directional signaling and co-expression of the RGS-RhoGEFs in common backgrounds has impeded the determination of their relative intracellular effects on serum signaling^16,29,31^. Further, how such regulation by the RGS-RhoGEFs affects HIPPO signaling has been mainly unexplored. In this study, changes in CTGF transcript levels were used as a sensitive and quantitative surrogate measure of the effects of the RGS-RhoGEFs on serum signaling. All evidence found that ArhGEF12 is the primary GEF that transmits serum signaling to control the activation of YAP1. Conversely, ArhGEF1 was found to suppress peak serum signaling in a cell state and cell type dependent manner through a selective GAP activity for Gα_13_. Further, a low level of ArhGEF1 was required to prevent basal signaling that otherwise caused cells to undergo extreme flattening and increased constitutive Yap1 activity. This points to Gα_13_ in these cells as the primary conduit for serum mediated activation of YAP1. Given the reported stable association of ArhGEF1 with Gα_13_ in cells^45^, it is also speculated that ArhGEF12 may preferentially mediate serum signaling by better displacing ArhGEF1 from Gα_13_ than ArhGEF11. Such a competition mechanism is supported by our observations that ArhGEF11 and ArhGEF12 are equally required for Yap1 activation in cells in which Gα_12/13_ are overexpressed or if ArhGEF1 expression was silenced.

In cells in tissues it is predicted that ArhGEF1 fine-tunes signaling by serum mitogens to regulate F-actin formation and YAP1 activity as cells transition into a stable monolayer. LPA and PAR promote cell invasion and proliferation during tissue regeneration and cancer cell spreading^52^. For instance, serum signaling promotes cells at the leading edge of a wound to activate actin stress-fiber dependent events that drives both adhesion and Yap1/TEAD related proliferation^8^. The specific effects of ArhGEF1 to cells at intermediate density suggests that it primarily functions at the point following cell proliferation where cells are increasingly contacting each other. ArhGEF1 may, at this point, inhibit serum signaling to reduce stress-fiber associated adhesion that promotes tensile forces that activate Yap1. This role is supported by the observations that cells with complete ArhGEF1 silencing remain highly flattened and do not assemble into the typical “cobblestone” monolayer seen for control MCF7 cells. Intriguingly, as cells form intercellular contacts, ArhGEF1 is reported to redistribute to cell junctions in breast cancer cells where it stimulates RhoA activation of diaphanous to promote cortical F-actin formation^46,47^. Thus, ArhGEF1 may promote epithelial cell cohesion by both initially downregulating stress fiber associated adhesion and then by re-localizing to cell–cell contacts to stimulate cortical F-actin formation for junctional maturation.

The selective ability of ArhGEF1 to basally deactivate signaling by Gα_13_, but not Gα_12_ also suggests that it is part of a “fail-safe” mechanism to prevent extreme cellular outcomes such as cancer or senescence. Mice with a genetic inactivation of Gα_13_ but not mice inactivated for Gα_12_ experience defects in cell survival and growth that results in embryonic lethality^48^. This indicates that activation of Gα_13_ is especially important in the pro-growth activities of serum mitogens. Because YAP1/TEAD activity is highly linked to the malignancy of breast and other types of cancer^49,50^, ArhGEF1 potentially prevents Gα_13_ from contributing to YAP1 mediated pro-cancer phenotypes. Conversely, comparison of the effects of differential ArhGEF1 silencing revealed that a small fraction of its activity was sufficient to prevent constitutively elevated RhoA activity that produced extreme cell flattening. These effects are reminiscent of the phenotypes of senescent cells produced by chronic mitogenic signaling such as extended RAS signaling in a p53 wild-type background^51^ and during chronic growth factor signaling under activated Rb^52^. Thus, ArhGEF1 by preventing serum mitogens such as LPA or PAR from having overly dominant effects may also prevent the acquisition of senescence.

## Experimental procedures

### Cell culture

BT474 (ATCC), Human Embryonic Kidney (HEK) 293 T cells (ATCC), MDA-MB-231 cells (ATCC) and Michigan Cancer Foundation 7 (MCF7) cells (L. Malkas, City of Hope) were cultured in Dulbecco’s modified Eagle’s medium (DMEM) with high Glucose and 10% Fetal Bovine Serum (FBS) and 0.5% Hyclone (Thermo SV300010) (v/v). All cells lines were grown on plastic dishes except MDA-MB-231 cells which were grown in the low mechanical loading background of growth factor depleted Matrigel (Corning). MCF7, BT474s and HEK293Ts cells were transfected with the PEI (Aldrich) as described^5^. Serum add back consisted of DMEM (high glucose) with 10% FBS. GPCR agonists were added to cells in DMEM as the following preparations: LPA 1 μM with 1 μg/μl of fatty acid free bovine serum albumin carrier, TFLLR-NH2 at 10 μM and 2-furoyl-LIGRLO-NH_2_ at 5 μM.

### Antibodies

Antibodies for immunoblot were purchased from the companies and used at dilutions as follows: Flag (Sigma, F3165) 1:10,000, 9E10 (Developmental studies hybridoma databank) 1:1000, p115^16^ 1:1000, PDZ RhoGEF (Santa Cruz, sc-67023) 1:1000, LARG RhoGEF (Santa Cruz, sc-25638), 1:1000 RhoA (Sant Cruz sc-179), 1:2000 snRNP70 (Santa Cruz sc-9571), GAPDH (Millipore, MAB374) 1:10,000, pYAP1 (Cell Signaling, #4911) 1:1000, YAP1 (Abnova, H00010413-M01) 1:1000. For immunostaing PDZ RhoGEF (Bethyl, A301-952A) 1:1000, LARG RhoGEF (Bethyl A301-959A) 1:1000, 1:250 YAP1 (Abnova, H00010413-M01). For IP, 1:100 Gα_13_-GTP (East Biosciences 26902).

Bradykinin and *PAR-activating peptides:* Bradykinin peptide RPPGFSPFR (Tocris Biosciences #3004) used at 10 nM. The receptor-selective PAR-activating peptide 2-furoyl-LIGRLO-NH_2_ for PAR2 was prepared by solid phase synthesis, purified to > 95% purity by HPLC and verified by mass spectral analysis by the University of Calgary peptide synthesis facility (peplab@ucalgary.ca). Cells were exposed to optimally active concentrations of these agonists (2 to 10 μM).

### Recombinant DNA

The RhoGAP and RhoGEF containing plasmids for this study were subcloned in^53^ and parent cDNAs are listed in Supplemental Table 1. The Gα_13_ (QL) mutant was originally described in^36^. Descriptions of synthesized DNA products (Primers and G-Blocks) are described in Supplemental Table 2. Ga Mission small hairpin silencing lentiviral vectors (Sigma): shArhGEF1 (TRCN0000033566, TRCN0000033568), shArhGEF11 (TRCN0000047465), and shArhGEF12 (TRCN0000298941, TRCN0000298942), shYAP1 (TRCN0000107266). Addgene vectors: Scramble shRNA (#1864), psRSV-Rev (#12253), pMDLg-RRE (#12251), and pCMV-VSVG (#8454).

### Immunoblot, immunoprecipitation and cell fractionation assays

Cells lysates prepared in RIPA buffer (50 mM Tris pH 8.0, 2 mM EDTA, 10% Triton-100, 150 mM NaCl, 0.1% SDS) containing 1 mM NaVO_4_, 2 mM β-glycerol phosphate, and protease inhibitor cocktail (Sigma) were resolved by SDS-PAGE and transferred using GenieBlot. Images were developed by a LiCor Odyssey and quantified with ImageJ^54^. For immunoprecipitations, five million control or ArhGEF1 silenced MCF7 cells were lysed in 1 ml PLC buffer described in^5^ with 1 mM GTP and incubated for 2 h with 25 μl anti-Gα_13_-GTP antibody. After 30 min. incubation with protein–G sepharose, beads were washed three times in PLC buffer and then eluted by boiling in SDS sample buffer. Cytosol and Nuclear Fractions were prepared by lysing 4 M MCF7 cells at 70% confluence in 1 mL of 10 mM HEPES pH 7.9, 1.5 mM MgCl_2_, 10 mM KCl, and 0.05% IGEPAL for 10 min on ice. Cytosol represents the supernatant from centrifugation at 3000 RPM for 10 min. The pellet was washed and resuspended in RIPA. Ten micrograms of protein for each condition was resolved by SDS-PAGE for immunoblot analysis.

### Dual Luciferase reporter assay

400,000 BT474 cells were seeded into 17.5 mm plates and then cultured for 18 h before being transfected with 0.04 μg TK-Renilla, 0.05 μg TEAD4-β-Galactosidase (GAL), 0.4 μg GAL-Luciferase Reporter, 0.23 μg 3X-FLAG YAP1, and 1 μg of the indicated RhoGEF or RhoGAP plasmid. C3 exotransferase was expressed by transient expression of a pCMV5 vector encoding this ORF. Lysates were prepared from cells 18 h after transfection, and luciferase activity was measured by Dual-Luciferase Assay System (Promega). Error bars represent standard deviation of mean. All p-values computed by 1-way Anova with Bonferroni post-hoc test unless otherwise stated.

### RhoA activity assay

The active GTP-bound form of RhoA and total RhoA were measured in a 96-well format with each condition in biologic quadruplicate by the G-Lisa system (Cytoskeleton Inc.) as described in the manufacturer’s protocol.

### Immunofluorescence imaging

Cells were plated onto acid washed coverslips at the indicated densities for 24 h and then switched to media with the indicated serum condition for 24 h. Treatments were added before cells were fixed and developed by immunostaining, Phalloidin-594 (Santa Cruz, sc-363795) and/or DAPI (Sigma, D9542). Slides mounted in ProLong Gold (Life Technologies, P36930) were imaged with Grid-Confocal microscopy (Zeiss AxioObserver) using a 63X oil Plan Apochromat objective.

### Brightfield imaging

Cells were plated in 3.5 cm or 6 cm dish and 40 × phase contrasted images were captured with a Zeiss AxioObserver microscope.

### RNA isolation and real-time quantitative PCR

cDNA synthesis used 5 µg total RNA extracted with Tri Reagent (Sigma), random hexamers and Superscript (ThermoFisher). Real-time (rt) quantitative (q) PCR (Supplemental Methods) used SensiMix SYBR NO-ROX (Bioline) in an Eppendorf Realplex2. Relative mRNA Levels were calculated by the 2^−ΔΔCT^ method as fold-change from control which in all cases was normalized to one. Fold Change in Gα induction of CTGF mRNA was computed as ((G-protein 2^−ΔΔCT^ with shArhGEF)/(control with shArhGEF 2^−ΔΔCT^)) – ((G-protein with shScramble 2^−ΔΔCT^)/(control with shScramble 2^−ΔΔCT^)).

## Supplementary Information

Supplementary Information.
