# Supplementary material for: The RGS-RhoGEFs control the amplitude of YAP1 activation by serum
Source: Sci Rep. 2021 Jan 27;11:2348. doi: 10.1038/s41598-021-82027-4 (PMC7841162; doi:10.1038/s41598-021-82027-4)
Supplement: Supplementary file 1 — Supplementary Information. [file 41598_2021_82027_MOESM1_ESM.docx]

­­­­**SUPPLEMENTAL FILES**

The RGS-RhoGEFs Control the Amplitude of YAP1 Activation by Serum

**Brandon S. Lane, Brigitte Heller, Morley D. Hollenberg, Clark D. Wells**

**TABLE OF CONTENTS**

Supplemental Methods 2

Supplemental Tables 2

Supplemental Figures 5

Supplemental References 14

Raw Images Used in Manuscript 15

**Supplemental Methods**

***Transfection of siRNA***. MCF7 cells were reverse transfected in antibiotic-free Optimem (1 mL/well) using Dharmafect and the indicated siRNA (final concentration of 30 nM after plating) before being plated at 3 x 10^5^, 6 x 10^5^ or 2 x 10^6^ cells per 35 mm well. After overnight incubation (12 hours), media was replaced with complete media and cell were grown for an additional 12 hours. Cells were then serum starved for 24-hours (grown in serum free optimum) before being treated with media alone or media containing 10 % serum for 30 minutes. Cells were then harvested for either protein or mRNA extraction.

***CTGF Reporter Cloning.*** The wild-type 4.5 kb CTGF promoter and a variant with the 1^st^ SRF site mutated described in (1) was obtained from Dr. Margarete Goppelt-Struebe. Both CTGF promoter variants were inserted into the MCS of pGL3-basic vector at the 5’ KPNI and 3’ XhoI restriction enzyme sites. A The MLUI site ~4000 bp from the 3’ end was disrupted by site directed mutagenesis and a 5’ KPNI site was introduced for insertion into the MCS. This CTGF promoter variant is the long form encompassing ~3.9 kb. The short ~700 bp CTGF promoter was generated by digestion with MLUI and XhoI, which was then ligated into the basic-pGL3 vector. Site directed mutagenesis of SRF binding sites (2-3) (TAT🡪CCC) (1) and TEAD binding sites (1-2) (AAT🡪GCG) (2) were conducted by the Stratagene method using the primers listed in Supplemental Table 2. A gBlock was synthesized by IDT that contained the desired mutations for the TEAD 5-7 binding sites. This gBlock was inserted into the short and long promoter using a 5’MLUI site and a 3’ SacII restriction enzyme site. All clones were sequence verified.

**Supplemental Tables**

*Supplemental Table 1: cDNA Expression Constructs of GEFs and GAP*

| **Protein** | **Human or Mouse** | **Accession #** | **V#** |
| --- | --- | --- | --- |
| ALS2 | Human | BC031479 | V2681 |
| ARAP1 | Human | AB018325 | V2713 |
| ARHGAP 1 | Human | BC018118 | V2661 |
| ARHGAP 11A | Human | D87717 | V2693 |
| ARHGAP 17 | Mouse | BC003259 | V2744 |
| ARHGAP 2 | Human | BC011393 | V2649 |
| ARHGAP 20 | Human | AB037845 | V159 |
| ARHGAP 25 | Human | D29642 | V2694 |
| ARHGAP 26 | Human | AB014521 | V2708 |
| ARHGAP 28 | Human | BC033668 | V2687 |
| ARHGAP 31 | Human | AB033030 | V2721 |
| ARHGAP 35 | Human | AB051509 | V2732 |
| ARHGAP 4 | Human | D50921 | V2695 |
| ARHGAP 9 | Human | BC006107 | V2745 |
| Arhgap12 | Mouse | BC024633 | V2667 |
| Arhgap15 | Mouse | BC034881 | V2688 |
| Arhgap18 | Mouse | BC030858 | V2678 |
| Arhgap24 | Mouse | BC027070 | V2672 |
| ArhGAP29 | Mouse | BC040387 | V2691 |
| ARHGAP39 | Human | AB051510 | V2733 |
| ARHGAP8 | Mouse | BC010306 | V2647 |
| ARHGEF 1 | Human | BC034013 | V2747 |
| ARHGEF 10 | Human | AB002292 | V2699 |
| ARHGEF 10L | Human | AB046846 | V2731 |
| ARHGEF 11 | Human | AB002378 | V2703 |
| ARHGEF 12 | Human | AB002380 | V2704 |
| ARHGEF 15 | Human | AB020722 | V2716 |
| ARHGEF 16 | Human | BC002681 | V2633 |
| ARHGEF 17 | Human | AB002335 | V2701 |
| ARHGEF 2 | Human | AB014551 | V2709 |
| ARHGEF 25 | Human | BC012860 | V2655 |
| ARHGEF 4 | Human | AB029035 | V2718 |
| ARHGEF 5 | Human | BC010046 | V2645 |
| ARHGEF 6 | Human | D25304 | V2692 |
| ARHGEF 7 | Human | D63476 | V2696 |
| ARHGEF 9 | Human | AB007884 | V2706 |
| ARHGEF39 | Human | BC033666 | V2686 |
| ARHGEF8 | Human | BC010285 | V2646 |
| DNMBP | Human | AB023227 | V2746 |
| DOCK 4 | Human | AB018259 | V2710 |
| DOCK6 | Human | AB037816 | V2727 |
| DOCK7 | Human | AB051558 | V2734 |
| Ect2 | Mouse | BC025565 | V2668 |
| Fam13b | Mouse | BC031465 | V2680 |
| Farp1 | Mouse | BC030329 | V2676 |
| FARP2 | Human | AB018336 | V2714 |
| FGD1 | Human | BC034530 | V290 |
| FGD2 | Human | BC023645 | V2665 |
| FGD6 | Human | AB037783 | V2725 |
| Fgd6 | Mouse | BC026860 | V2671 |
| HMHA1 | Human | D86976 | V2613 |
| Inpp5B | Mouse | BC028864 | V2674 |
| INTERSECTIN 2 | Human | AB033082 | V2722 |
| NGEF | Human | BC031573 | V2682 |
| P-REX1 | Human | AB037836 | V2728 |
| Plekhg6 | Mouse | BC026778 | V2670 |
| RACGAP1 | Human | BC032754 | V2685 |
| RALBP1 | Human | BC013126 | V2656 |
| SH3BP1 | Human | BC008282 | V2642 |
| SRGAP3 | Human | BC039300 | V2705 |
| Stard13 | Mouse | BC027830 | V2673 |
| STARD8 | Human | D80011 | V2697 |
| SYDE1 | Human | BC029926 | V2675 |
| TAGAP | Human | BC015859 | V2659 |
| VAV1 | Human | BC013361 | V2657 |

*Supplemental Table 2: Primer sets and gBlocks used in cloning CTGF Promoters*

| **Promoter** | **Mutations** | **Sense** | **Antisense** | **gBlock** |
| --- | --- | --- | --- | --- |
| Short | WT | X | X |  |
| Short | TEAD Mut | X | X | 3x TEAD gblock |
| Short | TEAD/SRF Mut | CCTACCCAGGATGTATGTCAGTGGACAGAACAGGGCAAACTTATT | CATCCTGGGTAGGAAGTAGGTAGCTGAAAGAGGCAAACAGCAG | 3x TEAD gblock |
| Short | SRF Mutat | CCTACCCAGGATGTATGTCAGTGGACAGAACAGGGCAAACTTATT | CATCCTGGGTAGGAAGTAGGTAGCTGAAAGAGGCAAACAGCAG |  |
|  |  |  |  |  |
| Long | WT | X | X |  |
| Long | 1st SRF Mut | X | X |  |
| Long | 1st & 2nd SRF Mut | CATACCTGGGATAGCTTGGTAAACAGGACTCAGTGGCCAGC | GCTATCCCAGGTATGTGTGTGTGTCTACTAGGGCATCATTTGTACTGG |  |
| Long | 1, 2, and 3 SRF Mutant | CCTACCCAGGATGTATGTCAGTGGACAGAACAGGGCAAACTTATT | CATCCTGGGTAGGAAGTAGGTAGCTGAAAGAGGCAAACAGCAG |  |
| Long | 1, 2, and 3 SRF and TEAD Mutant | GAGGGCGGCGAGGGCGGTCCCTGTTTGTGTAGGAC | GGGACCGCCTCGCCGCCCTCC | 3x TEAD gblock |
| Long | WT TEAD Mutants | GAGGGCGGCGAGGGCGGTCCCTGTTTGTGTAGGAC | GGGACCGCCTCGCCGCCCTCC | 3x TEAD gBlock |

**Supplemental Figures**


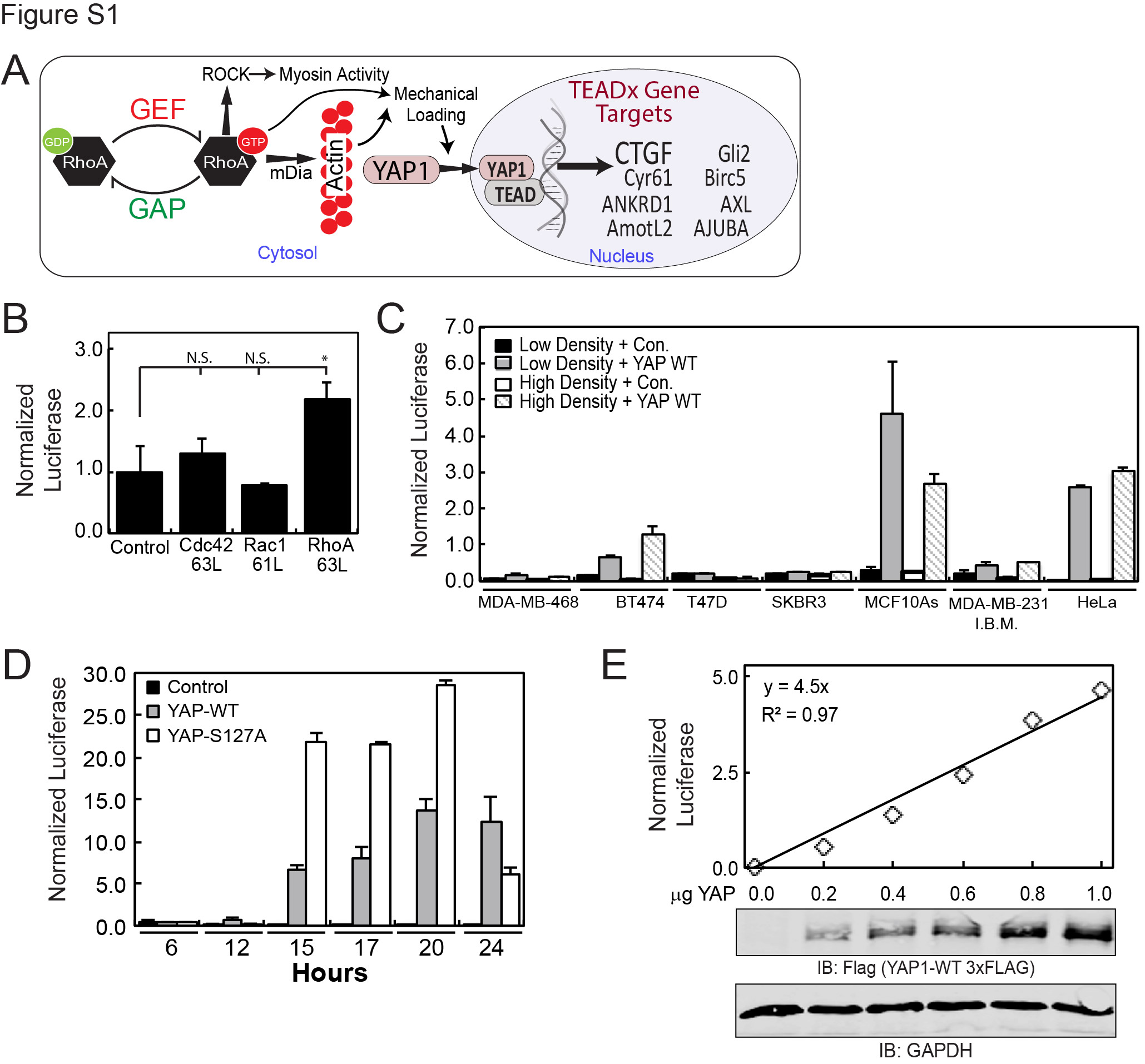


**Figure S1. Screen for RhoGEFs and RhoGAPs that modulate TEAD dependent Transcription. A.** Cartoon depicting the modes by which RhoA regulates filamentous actin accumulation and mechanical loading that is necessary for the nuclear translocation of YAP1 where it activates TEAD dependent transcription. **B.** The specificity of constitutive active RhoA-Q63L for the activation of YAP1 was reproduced here in BT474 cells expressing heterologous YAP1. BT474 cells were transfected with either constitutively active RhoA (Q63L), Rac1 (Q61L), or Cdc42 (Q63L) along with the TEAD reporter, YAP1 and TK-Renilla and then cultured for 18 hours. Cells were then lysed and luciferase activity was measured and graphed as normalized fold-change activity to control. **C**. The indicated cell lines were plated at high density (400,000 cells) or at low density (200,000 cells) into 35 mm dishes. After 24 hours, cells were transfected with plasmids encoding wild-type YAP1 or a control plasmid in combination with the TEAD4-Gal coupled 5xGal4-luciferase reporter and the TK-Renilla normalization plasmid. After 18-hours, cells were lysed and the relative luciferase activity was measured using the dual luciferase kit (Promega). Normalized luciferase activity represents firefly activity over Renilla activity. **D.** BT474 cells were transfected with wild-type flag-tagged YAP1, a flag-tagged mutant YAP1 (S127A), or control plasmid in combination with the TEAD4-Gal coupled 5xGal4-luciferase reporter as well as the TK-Renilla plasmids. Replicates were lysed and luciferase activity was measured at the indicated times and graphed as normalized activity **E.** BT474 cells were transfected with the indicated micrograms of flag-tagged wild-type YAP1 in combination with the TEAD-Gal coupled 5xGal4-luciferase reporter and TK-Renilla plasmids. After 18 hours, cells were lysed and luciferase activity was measured by the dual luciferase system (Upper panel). Cell lysates were also immunoblotted for the relative levels of flag-tagged proteins using the anti-flag (M2) antibody (Bottom Panel). Data represent mean values, error bars show standard deviation of mean. Variability unless indicated otherwise was queried by one-way ANOVA. * = p-value < 0.05 from contrasts identified by the Bonferroni Multiple Analysis Test; N.S. = highlighted non-significant contrasts. Raw Images for all immunoblots in supplementary figures are shown in Fig. S7.

**
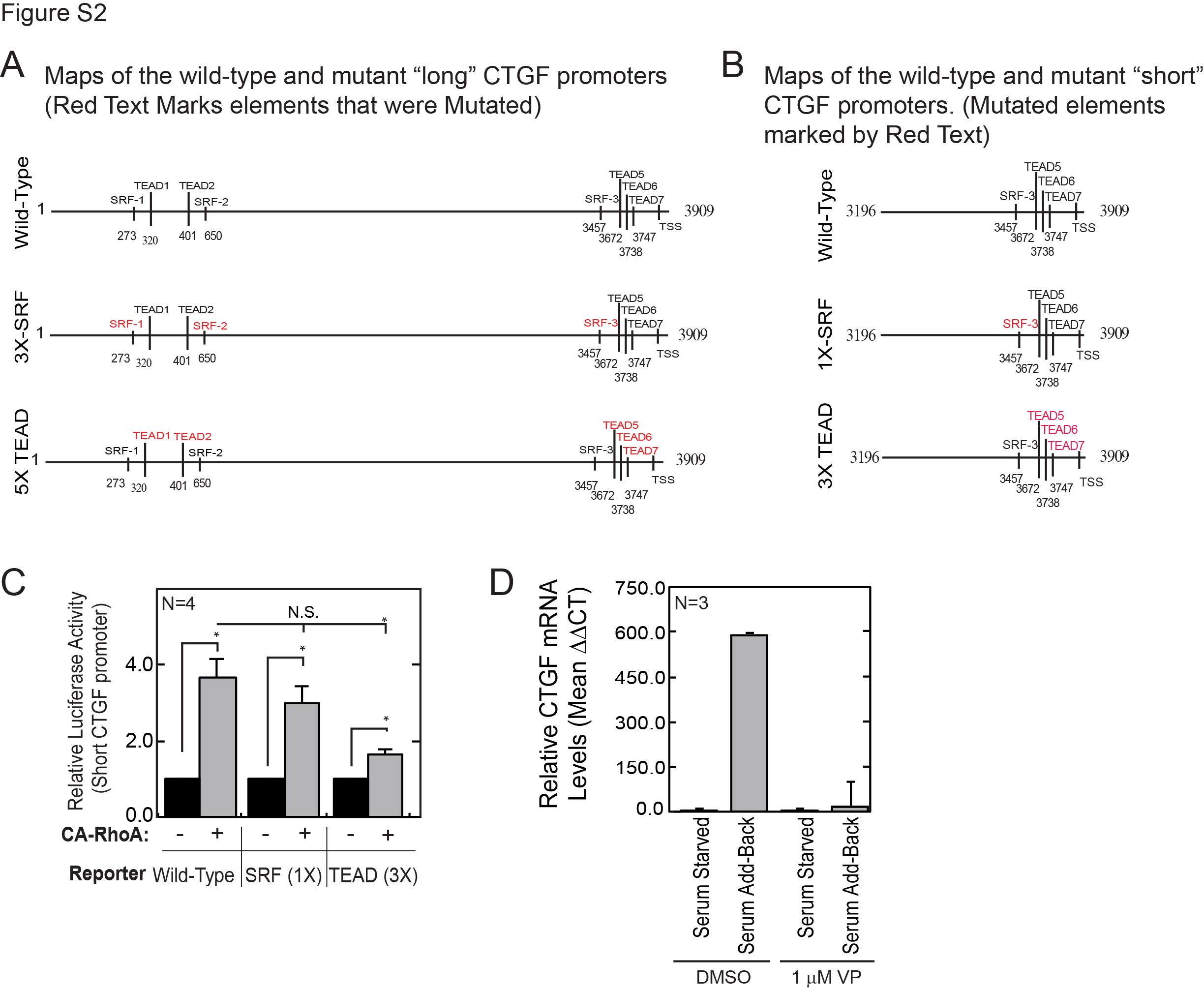
**

**Figure S2.** **CTGF Reporter Construct Cloning and Biological Replicates of RGS Domain Overexpression**. **A.** Schematics of the long form of the CTGF promoter (wild-type) showing the TEAD and SRF binding sites as well as the sites mutated in 3x-SRF long and 5x-TEAD long in red font. **B.** Schematic of the short forms of the CTGF wildtype short, 1x-SRF short, and 3x-TEAD short promoters in which the mutated binding sites are in red font. **C.** Fold-activation of the short 700 bp promoter of CTGF, the 1X SRF and 3X-TEAD mutant variants in the pGL3.1 reporter plasmid by expression of CA-RhoA (Q63L) versus a control in MCF7 cells. **D.** A graph of relative levels of CTGF transcription as measured by qrt-PCR of MCF7 treated with 1uM of Verteporfin following 24 hours serum starvation and then a 30-minute treatment with media containing either 0 % or 10% fetal bovine serum.

**
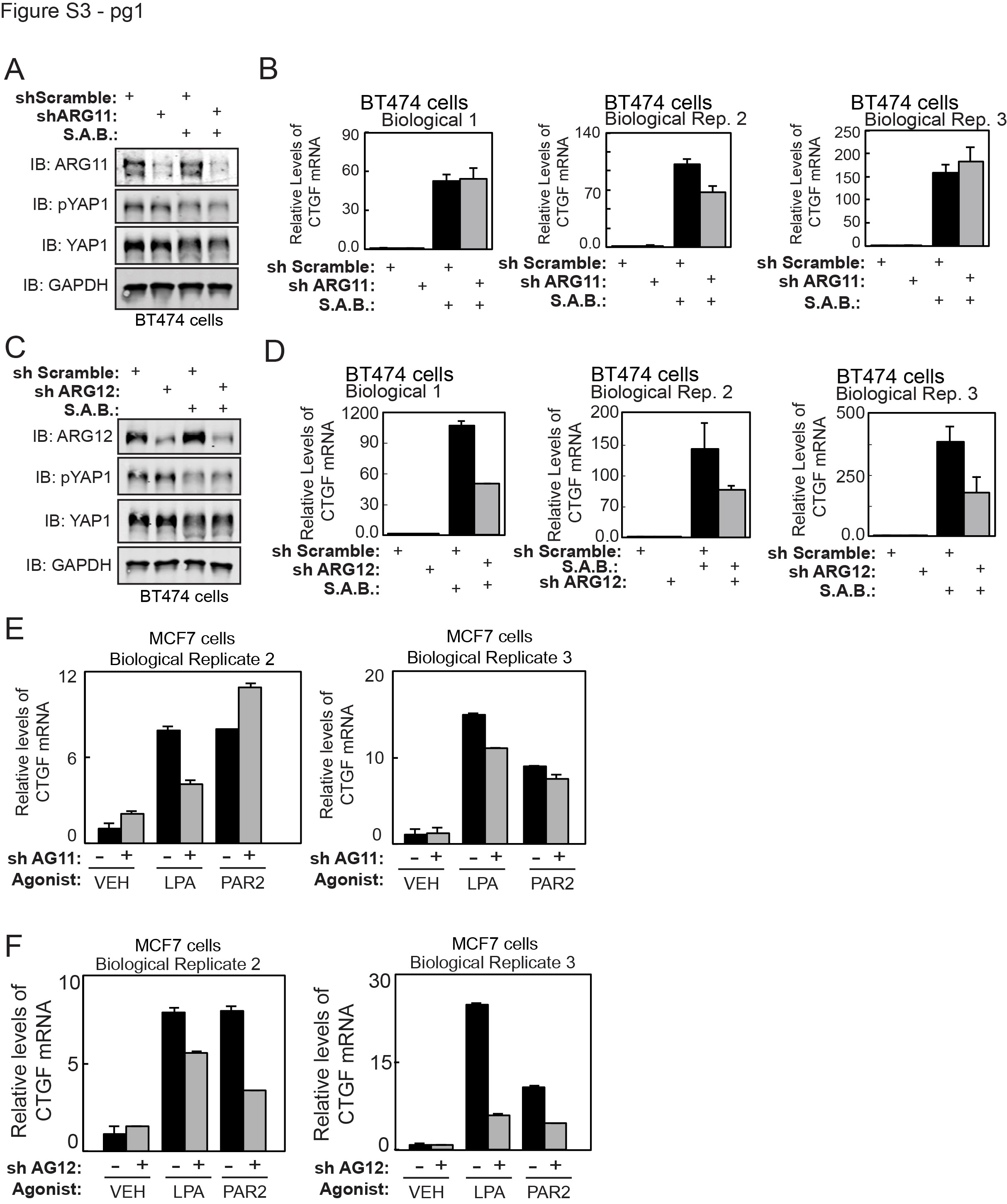
**

**­­­­**

**
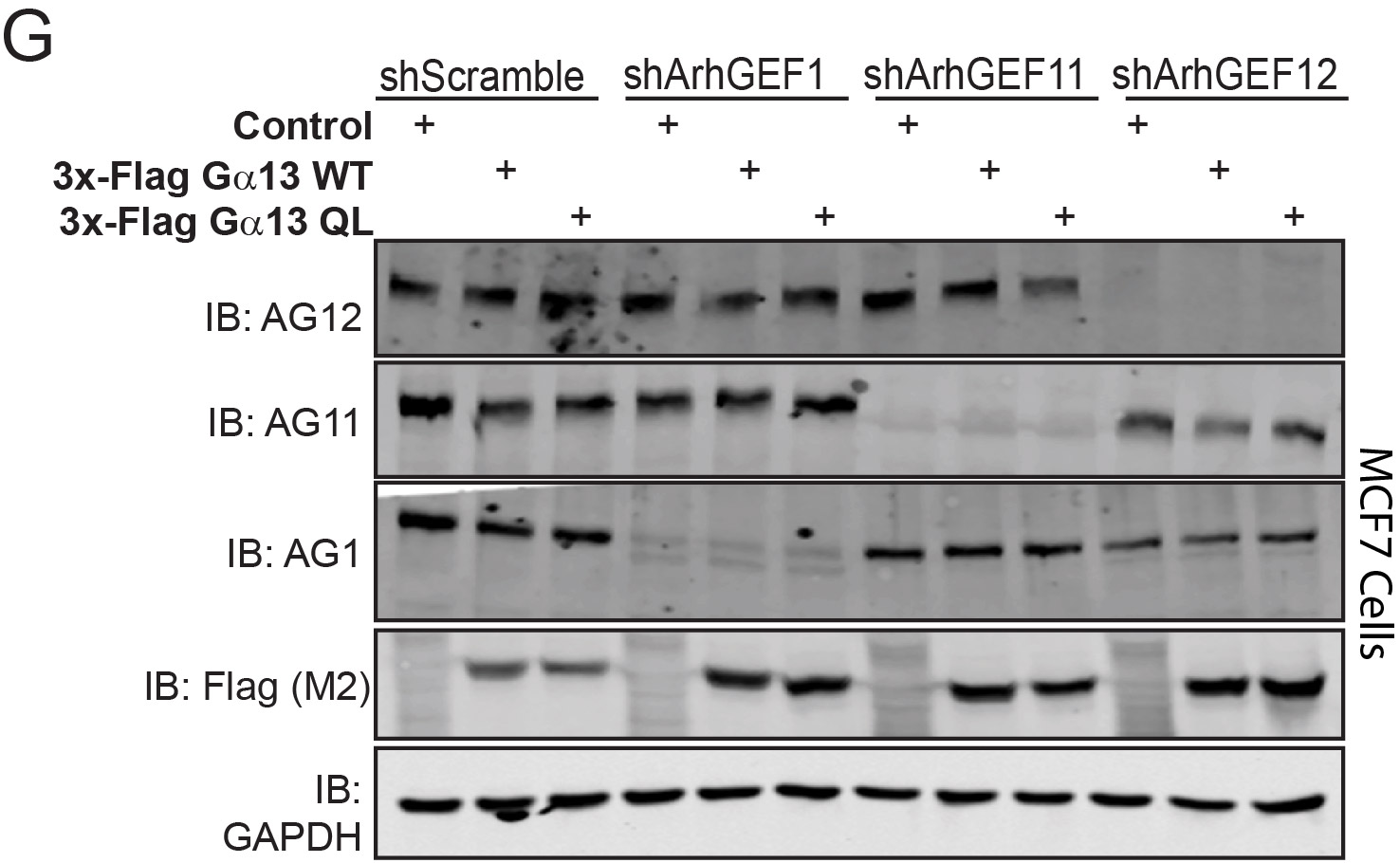
**

**Figure S3. Biological Replicates of ArhGEF11 and ArhGEF12 knockdown subjected to serum and individual agonist found in serum.**  **A.** Immunoblot analysis employing antibodies against ArhGEF11, phospho-S127 YAP1, total YAP1 and GAPDH on lysates prepared from BT474 cells transduced with lentivirus encoding either an shRNA scramble sequence or an shRNA against ArhGEF11. Prior to lysis, cells were serum starved for 24 h and then treated with media containing either 0 % or 10 % fetal bovine serum for 30 min. **B.** Three independent biologic replications of the fold change in CTGF mRNA as measured by qRT-PCR following 24-hour serum starvation and a 30-minute treatment with serum in BT474 cells transduced with lentivirus that drives expression of either shRNA encoding a scramble sequence that does not match any human sequence or shRNA against ArhGEF11 (shAG11). **C.** Immunoblot analysis employing antibodies against ArhGEF12, phospho-S127 YAP1, total YAP1 and GAPDH on lysates prepared from BT474 cells transduced with lentivirus encoding either an shRNA scramble sequence or an shRNA against ArhGEF12. Prior to lysis cells were serum starved for 24 h and then treated with 10 % fetal bovine serum for 30 min. **D.** Three independent biologic replications of measurements of the fold change in CTGF mRNA by qRT-PCR following 24-hour serum starvation and a 30-minute treatment with serum in BT474 cells transduced with lentivirus that drives expression of either shRNA encoding a scramble sequence that does not match any human sequence or shRNA against ArhGEF12 (shAG12). **E,F**. Biologic Replicates that measure the fold change in CTGF mRNA by qRT-PCR following 24-hour serum starvation and a 30-minute treatment with indicated agonist in ArhGEF11 silenced (shAG11) or ArhGEF12 silenced (shAG12) MCF7 cells compared to control. **G.** Immunoblot analysis of lysates prepared from MCF7 cells transduced with lentivirus that results in stable expression of shRNA against ArhGEF1, ArhGEF11 or ArhGEF12 in combination with lentivirus that stably expresses 3X-Flag tagged control, -Gα_13_, or – Gα_12_. Antibodies in this experiment were anti-ArhGEF1, -ArhGEF11, -ArhGEF12, Flag (M2) and GAPDH. These cells were used for experiments in Figures 3F, 6D, and S3 O-R.

**
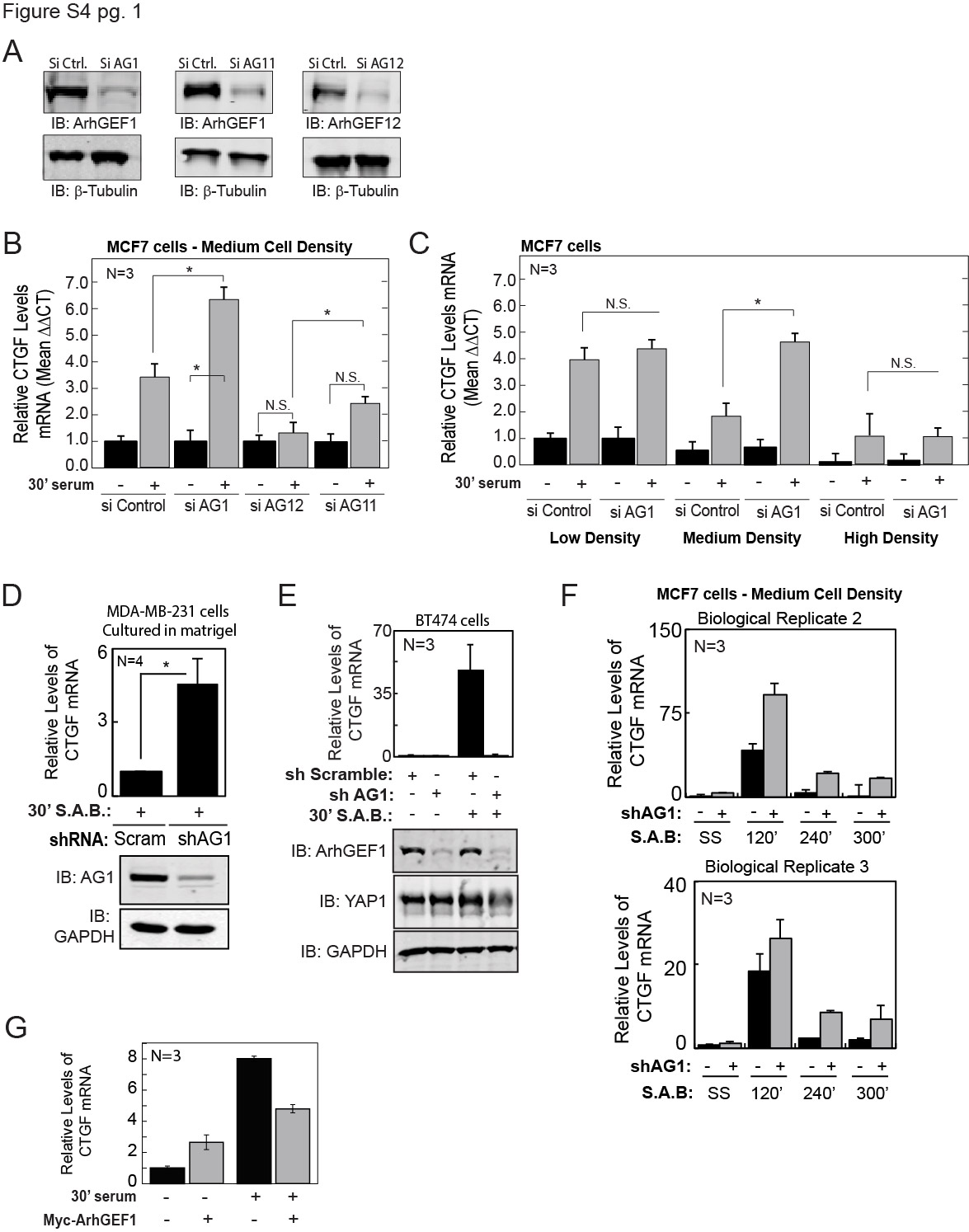
**

**
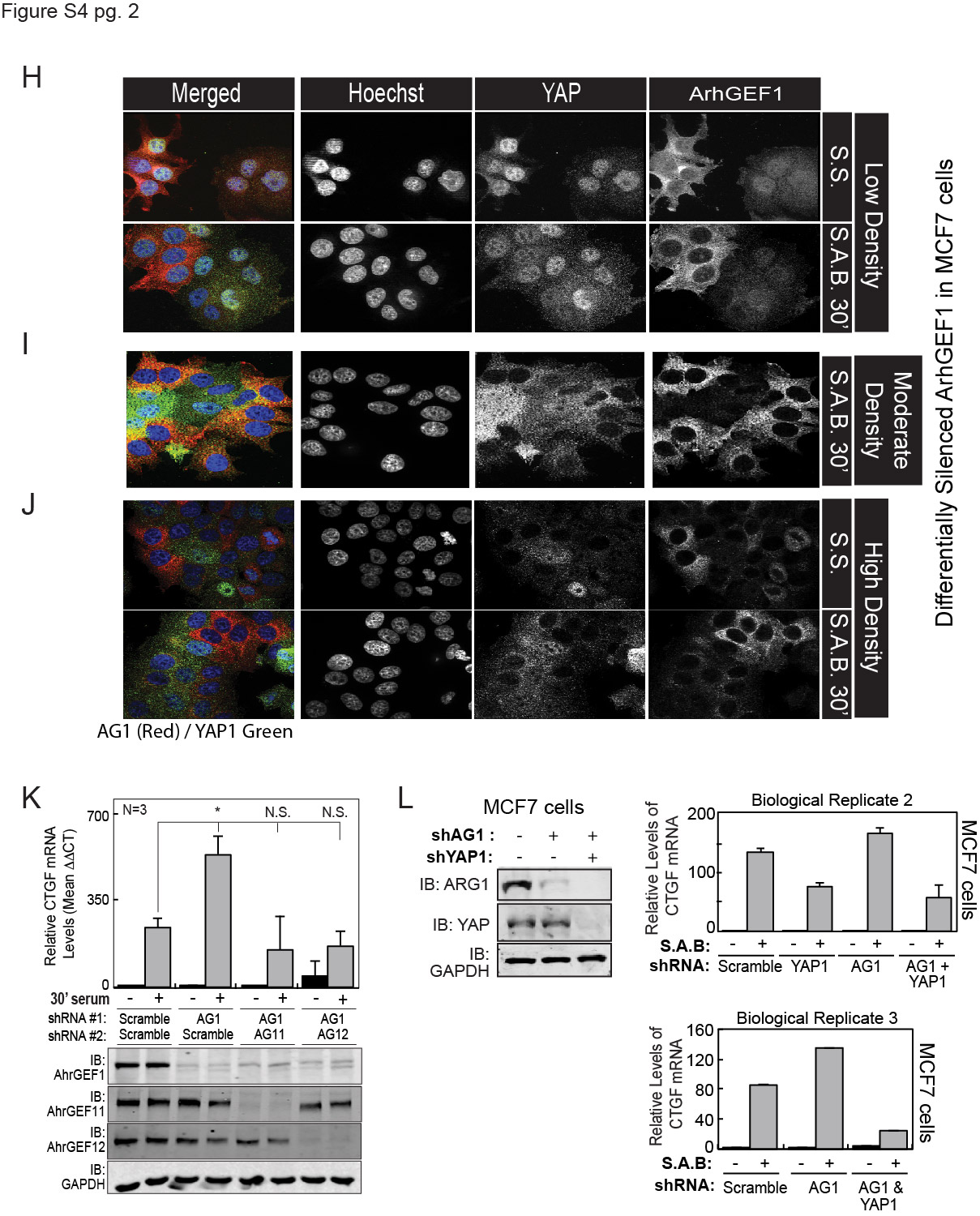
**

**Figure S4.** **Biological Replicates of ArhGEF1 knockdown subjected serum stimulation and density dependence of ArhGEF1 on CTGF transcription.** **A.** MCF7 cells (35 mm wells) were transfected by the Dharmafect method with Dharmacon, ON-TARGETplus non-targeting pool (D-001810), ArhGEF1 pool (L-009421), ArhGEF11 (L-010360) or ArhGEF12 pool (L-008480). Cells were grown for 16 hours, switched into serum free DMEM for 24 hours and then treated with media with or without 10 % serum for 30 minutes. Duplicate plates were lysed in RIPA for immunoblot analysis of control and silenced cells with the indicated antibodies or for **B.** cells were lysed with Trizol qRT-PCR analysis to measure the fold-change in CTGF transcript levels between conditions. **C.** MCF7 cells (35 mm wells) were reverse transfected by the Dharmafect method with Dharmacon, ON-TARGETplus non-targeting pool (D-001810) or ArhGEF1 pool (L-009421). Cells were plated at low, medium or high density and grown for 14 hours. Cells were then switched into serum free DMEM for 24 hours and then treated with media with or without 10 % serum for 30 minutes. Duplicate plates were lysed in Trizol for qRT-PCR analysis to measure the fold-change in CTGF transcript levels. **D.** Fold-change in CTGF transcript levels were measured by quantitative-real-time-PCR in MDA-MB-231 cells that were transduced with shRNA against ArhGEF1 or that expressed scramble shRNA (top panel). Immunoblot analysis of lysates prepared from replicate cells described in B using antibodies against ArhGEF1 and GAPDH. **E.** Fold-change in CTGF transcript levels were measured by q-rt-PCR in BT474 cells that were transduced with shRNA against ArhGEF1 or that expressed scramble shRNA (top panel). Immunoblot analysis of the cells described in B using antibodies against ArhGEF1, YAP1 and GAPDH. **F**. MCF7 cells transduced with lentivirus expressing either scramble control or shRNA against ArhGEF1 were serum starved for an additional 24 h and then treated with media with 0 % or 10 % serum for the indicated times for up to 5-hours. RNA was extracted with trizol from these cells, which was used to synthesize cDNA. The fold-changes in CTGF transcript levels were measured by q-RT-PCR. In lower panels, an immunoblot analysis from replicate lysates using antibodies against ArhGEF1 and GAPDH. **G**. MCF7 cells were transfected with a myc-control vector or with a vector that expresses Myc-ArhGEF1. Cells were grown for 15 hours following transfection before being transferred into DMEM lacking serum for 24 hours. Cells were then treated with DMEM with or without 10 % serum for 30 minutes before being lyzed in Trizol for mRNA extraction. Real-time quantitative PCR (Sybr Green) was then performed on cDNA synthesized from total mRNA. **H/I/J.** MCF7 cells with shControl of shArhGEF1 were plated at low, intermediate and high density on collagen coated cover slips. Cells were then fixed with PFA and immunostained with antibodies against ArhGEF1 and YAP1. In the merge panel at Left, DAPI (blue) was used to stain nuclei which is combined with the ArhGEF1 (Green) and YAP1 (Red) signals. **K**. Fold-change in CTGF transcript levels were measured by q-rt-PCR in MCF7 cells silenced for ArhGEF1 either alone or in combination with either ArhGEF11 or ArhGEF12 (top panel). After 24h or serum starvation cells were treated with media alone or media containing 10 % serum for 30 minutes. Immunoblot analysis of replicate plates of cells using antibodies against ArhGEF1, ArhGEF11, ArhGEF12 and GAPDH. **L.** Immunoblot of MCF7 cells that were silenced for ArhGEF1 alone or in combination with YAP1 (left panel). Antibodies used were against ArhGEF1 (ARG1), phospho-S127 YAP1 (pYAP), total YAP1 (YAP) and GAPDH. In the right two panels, the relative levels of CTGF transcript was measured by q-RT-CPR in MCF7 cells stably silenced for ArhGEF1 or YAP1 alone or in combination that were then serum starved before being treated with media alone or media containing 10 % FBS. Error Bars represent standard deviation of the mean. P-values computed by Anova with a Bonferroni post-hoc test. ** p-value < 0.01, ** p-value < 0.001.


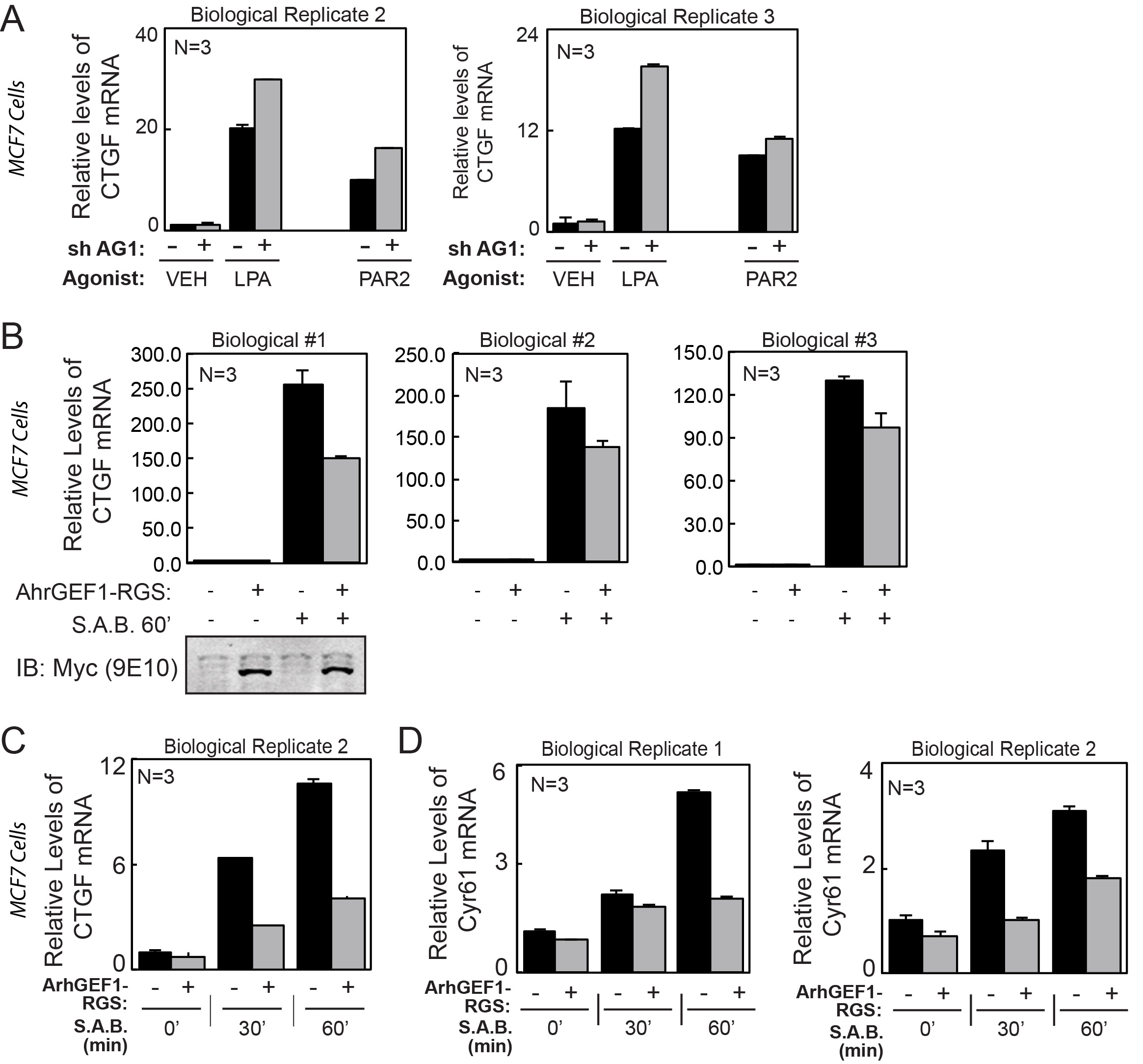


**Figure S5.** **Biological Replicates of ArhGEF1 knockdown subjected to individual agonist found in serum.**  **A.** The fold-change in CTGF transcript levels in cells stably silenced for ArhGEF1 or control that were also serum starved before a 30-minute treatment with vehicle or agonists against the LPA or PAR2 receptors. **B.** MCF7 were transfected with a control vector or a vector that expresses a myc-tagged RGS domain from ArhGEF1. Cells were then grown without serum for 24 H before treatment with serum for 60 min. Fold-change in CTGF transcript levels were measured by q-RT-PCR. An immunoblot of lysates prepared from replicate cells using and antibody against myc (9E10) is shown in the bottom panel . **C/D**. MCF7 were transfected with a control vector or a vector that expresses a myc-tagged RGS domain from ArhGEF1. Cells were then grown without serum for 24 H before treatment with serum for 0, 30, or 60 min. Fold-change in **C.** CTGF transcript levels or **D.** Cyr61 transcript levels were measured by q-RT-PCR.

Figure S6. Raw Images of Immunoblots in Main Manuscript


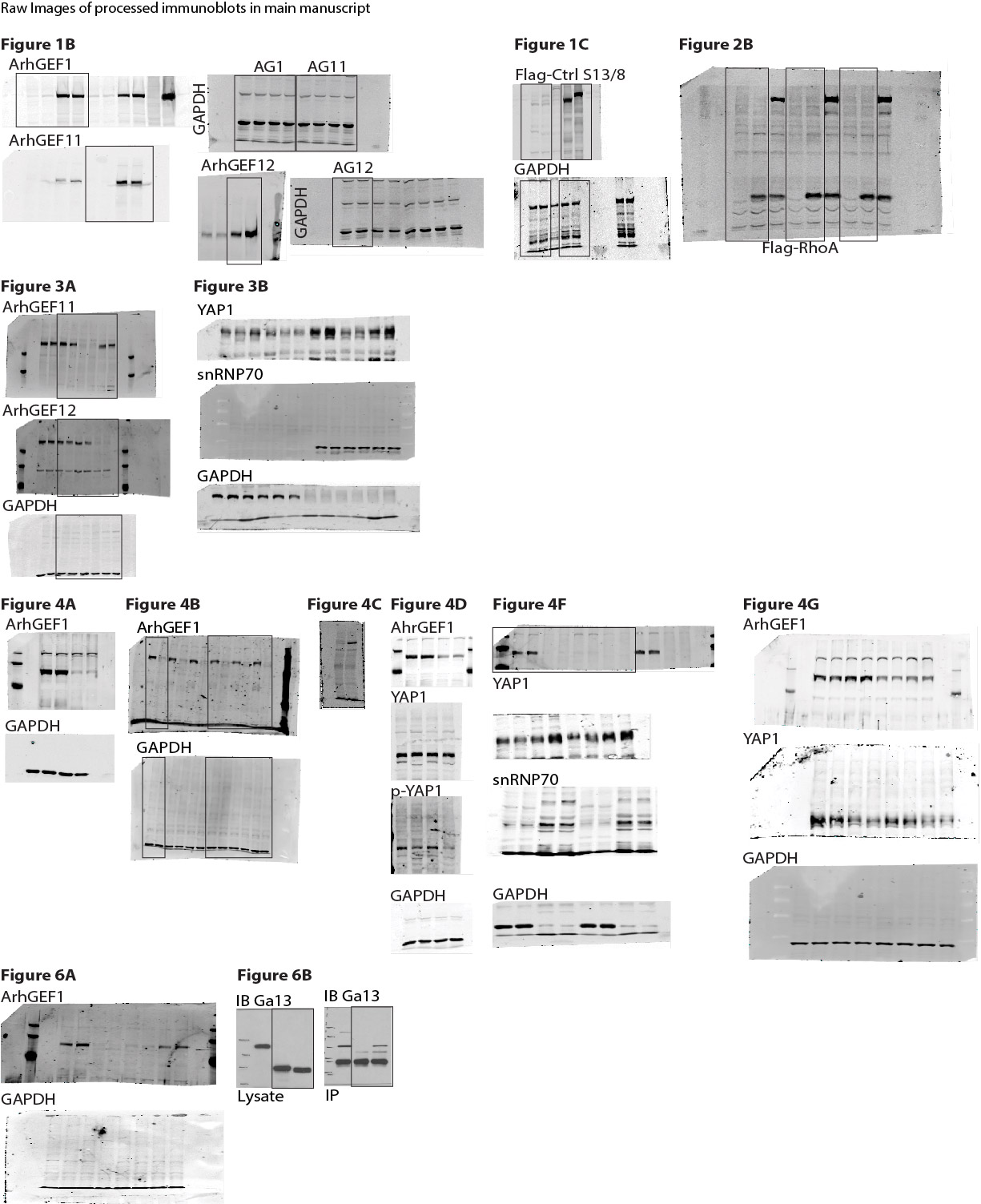


Figure S7. Raw Images of Immunoblots in Supplementary Material


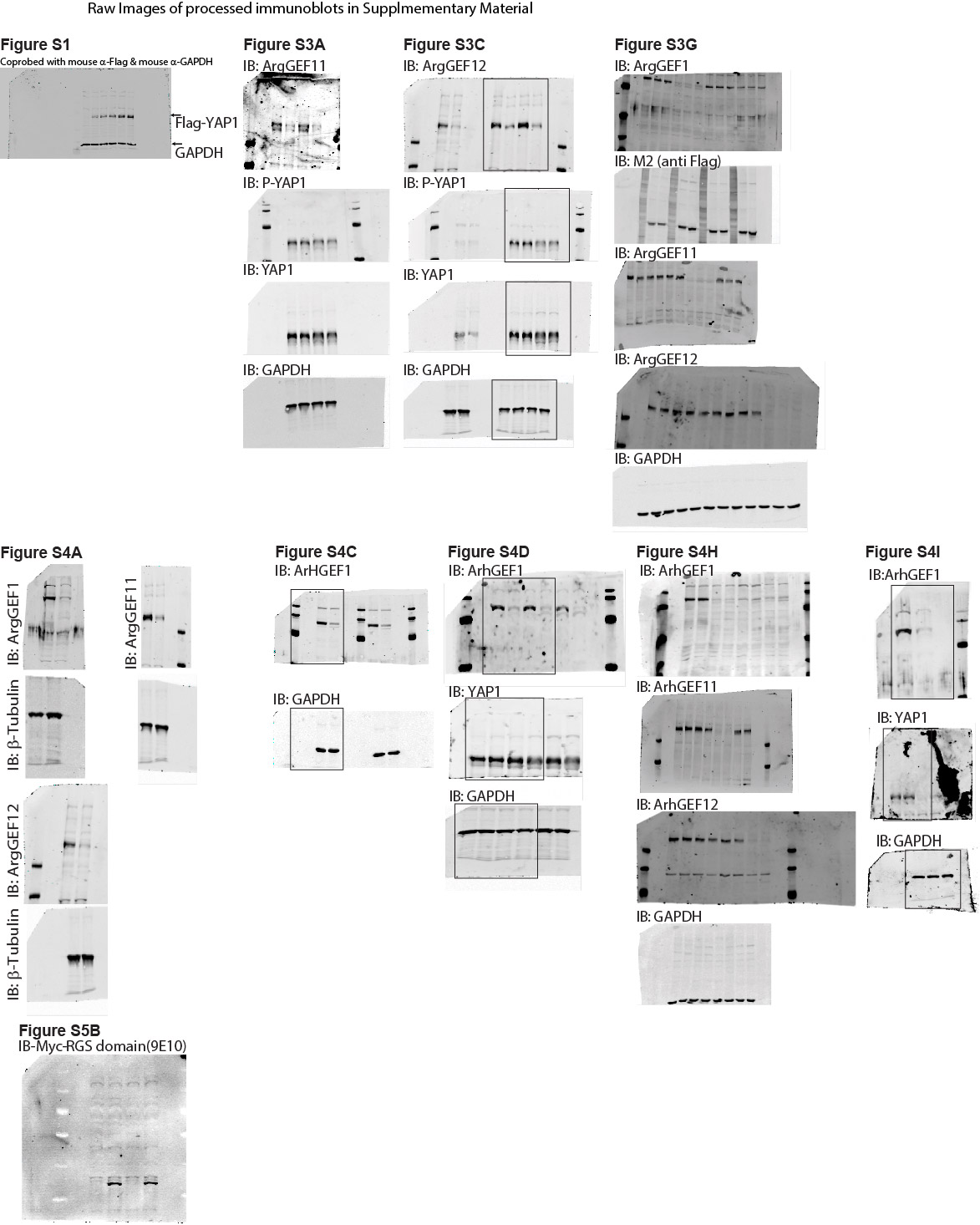


**REFERENCES**

1. Muehlich, S., Cicha, I., Garlichs, C. D., Krueger, B., Posern, G., and Goppelt-Struebe, M. (2007) Actin-dependent regulation of connective tissue growth factor. *American Journal of Physiology - Cell Physiology* **292**, C1732-C1738

2. Zhao, B., Ye, X., Yu, J., Li, L., Li, W., Li, S., Yu, J., Lin, J. D., Wang, C.-Y., Chinnaiyan, A. M., Lai, Z.-C., and Guan, K.-L. (2008) TEAD mediates YAP-dependent gene induction and growth control. *Genes & Development* **22**, 1962-1971
